# NIR‐II Emissive Persistent Neutral π‐Radical with Rapid Doublet Internal Conversion for Efficient Cancer Photothermal Theranostics

**DOI:** 10.1002/advs.202411733

**Published:** 2025-02-25

**Authors:** Qi Zhao, Chunxiao Wu, Yijian Gao, Jing Long, Wei Zhang, Yanan Chen, Yuliang Yang, Yu Luo, Yuxiao Lai, Houyu Zhang, Xiankai Chen, Feng Li, Shengliang Li

**Affiliations:** ^1^ College of Pharmaceutical Sciences The Fourth Affiliated Hospital of Soochow University Suzhou Medical College Soochow University Suzhou 215123 P. R. China; ^2^ State Key Laboratory of Supramolecular Structure and Materials College of Chemistry Jilin University Qianjin Avenue 2699 Changchun 130012 P. R. China; ^3^ Centre for Translational Medicine Research & Development Shenzhen Institute of Advanced Technology Chinese Academy of Sciences Shenzhen P. R. China; ^4^ Institute of Functional Nano & Soft Materials (FUNSOM) Jiangsu Key Laboratory for Carbon‐Based Functional Materials & Devices Soochow University Suzhou 215123 P. R. China

**Keywords:** conjugated oligomer, NIR‐II, photoacoustic imaging, photothermal therapy, radical

## Abstract

Organic radicals are considered prospective materials for near‐infrared (NIR) photothermal applications, however, sustainability remains the major obstacle of recently reported ionic radical photothermal agents. This work achieved robust sustainability on a series of neutral π‐radicals through rational design donor (D)–acceptor (A). With efficient doublet internal conversion, 10H‐spiro(acridine‐9,9′‐fluorene) (SFA)‐BTM presented strong NIR absorption extended to 1000 nm and efficient non‐radiative relaxation. SFA‐BTM nanoparticles (NPs) realized comparable NIR‐I photothermal conversion efficiency (PCE) and photoacoustic sensitivity. Also, the π‐radical NPs displayed NIR‐II emission and achieved high‐resolution whole‐body angiography for the first time by the NIR‐II bioimaging. Ultimately, the photothermal capabilities are confirmed in an orthotopic bone tumor model by effective ablation of cancer cells in vitro and inhibition of the deterioration of tumor in vivo. This research offers a new horizon in the conception and development of sustainable organic radicals for effective NIR‐II imaging and theranostics applications.

## Graphical abstract

Rational design of donor‐acceptor persistent neutral π‐radical develop ultra‐high photostability and thermostability, and simultaneously realize high‐resolution NIR‐II whole‐body angiography, photoacoustic imaging, and photothermal therapy of orthotopic bone tumors with good biosafety.

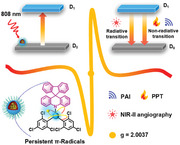

## 1 Introduction

Photothermal therapy (PTT) has emerged as a capable non‐invasive cancer therapy in recent years due to its significant spatiotemporal resolution and negligible adverse effects.^[^ ^1^ ^]^ Advanced therapeutics typically employ photothermal therapy agents (PTAs) to harvest the first near‐infrared (NIR‐I, 700–1000 nm) and second near‐infrared (NIR‐II, 1000–1700 nm) optical energy and generate in situ heat for efficient tumor elimination.^[^ ^2^ ^]^ In recent years, PTAs have developed rapidly and made significant progress, mainly including inorganic nanomaterials,^[^ ^3^ ^]^ inorganic–organic hybrid materials,^[^ ^4^ ^]^ polymer agents,^[^ ^5^ ^]^ supramolecular materials,^[^ ^6^ ^]^ and organic small molecule photothermal agents.^[^ ^7^ ^]^ The reported PTAs have mainly achieved acceptable NIR‐absorbing, effective photothermal generation, and relatively high photostability, thus providing sufficient tumor photothermal ablation in vivo.^[^ ^8^ ^]^ Although many advances have been received in the last few years, the lack of PTAs with potent photothermal conversion efficiency (PCE) and high bioavailability has led to its far less satisfactory therapeutic performance, which has seriously impeded the clinical translation of PTT.^[^ ^9^ ^]^ Thus, it is pressingly desirable to explore new photothermal materials, preferably with high PCE and biosafety.

Organic radicals are unique open‐shell materials with one or more unpaired electrons, and thus inherently exhibit characteristic physicochemical capabilities that the conventional close‐shell materials are missing.^[^ ^10^ ^]^ Due to their amazing capabilities, organic radicals are of fundamental research significance in the fields of physics, chemistry, and biomedicine. They also hold great promise in organic light‐emitting diodes (OLEDs),^[^ ^11^ ^]^ organic field‐effect transistors (OFET),^[^ ^12^ ^]^ molecular magnets,^[^ ^13^ ^]^ and batteries.^[^ ^14^ ^]^ Taking advantage of their tunable band gap and molecular structure, organic radicals are becoming powerful tools and platforms for chemical sensors, spin‐resonance probes, and fluorescence imaging. In particular, organic radicals exhibit naturally narrower energy gaps and are more conductive to form non‐radiative decay compared to closed‐shell materials. Thus, recently organic radicals have been widely utilized for NIR photothermal applications such as photothermal therapy^[^ ^6^, ^15^ ^]^ and solar‐thermal conversion.^[^ ^6^, ^16^ ^]^ Although organic radicals have been discovered for centuries and few methods have significantly improved their sustainability lately, achieving persistent and stable organic radicals remains a significant challenge.

**Scheme 1:**
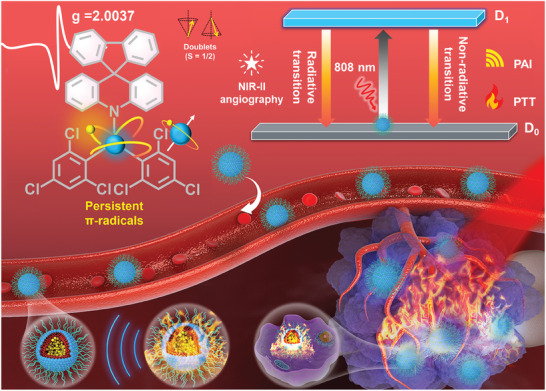
Design of D–A radical nanoparticles and their application in NIR‐II angiography and cancer photothermal theranostics. D_1_: the first doublet excited state, D_0_: doublet ground state, S: spin quantum number, NIR‐II: near‐infrared II, PAI: photoacoustic imaging, PTT: photothermal therapy.

In this contribution, we design donor‐acceptor (D–A) radicals with NIR absorption extended to 1000 nm with NIR‐II emission (**Scheme** **1**). The conjugated D–A small molecular radicals are engineered via conjugation coupling of electron‐withdrawing bis(2,4,6‐tirchlorophenyl)‐methyl radical (BTM) and electron‐donating acridine derivates. As demonstrated below, the designed D–A radicals show prominent NIR‐I absorption, NIR‐II emission properties, and ultrahigh stability even upon abominable light irradiation. Among these radicals, SFA‐BTM offers the most rapid internal conversion, favoring non‐radiative transition, and its nanoparticles (NPs) have a good photothermal conversion efficiency (PCE) of 49% and good stability. With the advantages, SFA‐BTM NPs for the first time achieved high‐resolution NIR‐II bioimaging of whole‐body blood vessels. Photoacoustic (PA) imaging‐guided NIR PTT indicates that SFA‐BTM NPs have excellent tumor ablation and bone repair effects in vitro and in vivo, particularly with good biosafety. Therefore, this study offers a practical molecular strategy for the development of persistent organic radicals with superior photothermal theragnostic of cancer in the NIR region.

## 2 Results and Discussion

### 2.1 Design, Synthesis, and Characterizations of Radical Molecular Materials

**Figure 1:**
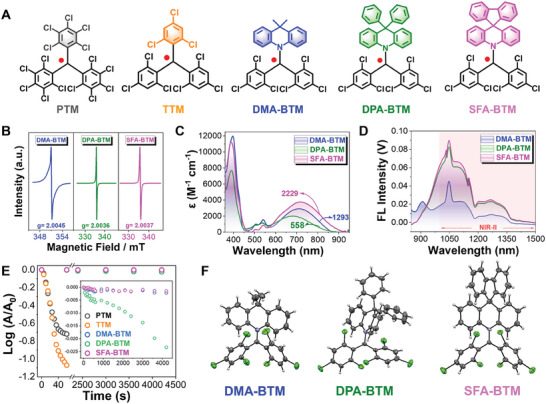
Molecular structures and characterizations of D–A radicals. A) Molecular structure of D–A radicals. B) EPR spectra measured of BTM radicals at solid state at 298 K. C) UV–vis–NIR absorption spectra of the three D–A radicals (50 µmol L^−1^) in cyclohexane. The extinction coefficient at 808 nm for each radical molecule is highlighted. D) Emission spectra of SFA‐BTM, DMA‐BTM, and DPA‐BTM in tetrahydrofuran (THF). These radical molecules with the same OD. The excitation light source is 808 nm laser. E) Time dependence of the photostability of D–A radicals in cyclohexane while irradiated with a 365 nm portable ultraviolet (UV) lamp. F) Crystal structures of DMA‐BTM, DPA‐BTM, and SFA‐BTM.

The D–A radical molecules were designed via conjugation coupling of carbon‐centralized bis(2,4,6‐tirchlorophenyl)‐methyl (BTM) radical with various electron‐donating segments (**Figure** **1**A). To explore suitable acridine derivates as D unit, three acridine derivatives SFA (abbreviation for 10H‐spiro(acridine‐9,9′‐fluorene)), DPA (abbreviation for 9,9‐diphenyl‐9,10‐dihydroacridine), and DMA (abbreviation for 9,9‐dimethyl‐9,10‐dihydroacridine) were smoothly conjugated into BTM core via the facile conjugately coupling reaction, yielding three D–A radicals DMA‐BTM, DPA‐BTM, and SFA‐BTM as isolated compounds with radically different configurations (Figures S1–S3, Supporting Information). The compounds were characterized by nuclear magnetic resonance (NMR) spectrometer, high‐resolution liquid chromatography mass spectrometry, matrix‐assisted laser desorption ionization‐time of flight mass spectrometry (Figures S4–S8, Supporting Information). As NMR characterization is inapplicable to free radicals, X‐ray crystal diffraction was employed to complementarily verify exact structures of the three radicals (Figure 1F; Tables S1–S3, Supporting Information). The central carbon atoms of these D–A radicals conjugated with the nitrogen atoms of acridine fragments via *sp^2^* hybrids for forming coplanar structures with a bond angle of nearly 120°. As a result of the steric hindrance of the Cl atoms, the D–A radicals show twisted propeller‐like shapes in configuration (Tables S4–S6, Supporting Information), enhancing their inertness to oxygen and water. To further decipher the chemical features of these D–A radicals, electron paramagnetic resonance (EPR) spectroscopy was employed to characterize the spinning resonance performance of these D–A radical molecules. As shown in Figure 1B, all D–A radical molecules exhibit a similar profile of one unpaired electron, substantially indicating their typical radical features. These results suggest that the molecular design and synthesis have successfully realized the D–A radicals.

Then, the UV–vis–NIR absorption spectra of D–A radicals were systematically investigated and shown in Figure 1C. DMA‐BTM, DPA‐BTM, and SFA‐BTM show typical maximum NIR absorption peaked at 710, 683, and 721 nm with the extinction coefficient of 2916, 2001, and 3747 m ^−1^ cm^−1^ in cyclohexane, respectively. The NIR absorption of D–A radical is associated with intramolecular charge transfer. The emission spectra and the corresponding relative fluorescence quantum yields of the D–A radicals are depicted in Figure 1D and Figure S9 (Supporting Information), respectively. These data collectively demonstrate that all D–A radicals show NIR‐II emission in tetrahydrofuran under laser excitation at 808 nm. According to time‐dependent density functional theory (TD‐DFT) calculation results (Tables S7–S10, Supporting Information), the maximum absorption in these D–A radicals is mainly supplied by the electronic transition from the highest β occupied orbitals to the singly unoccupied molecular orbitals (SUMO), forming the first doublet excited state. That is 147β to 148β for DMA‐BTM, 179β to 180β for DPA‐BTM, and 178β to 179β for SFA‐BTM. To test the photostability of three‐D–A radicals, the degradation velocity in absorption intensity was measured upon 365‐nm UV lamp irradiation in a sealed cuvette. By contrast, the frequently used radicals perchlorotriphenylmethyl (PTM) and tris(2,4,6‐trichlorophenyl)methyl (TTM) were chosen as comparators. As illustrated in Figure 1E and Figure S10 (Supporting Information), PTM and TTM show rapid degeneration in the absorption intensities and almost disappear after 40 s irradiation, which matches well with the reported results.^[^ ^11^, ^17^ ^]^ It should be noted that only negligible degradation is observed in these D–A radicals, and their radical properties were almost unchanged before and after irradiation (Figure S11, Supporting Information). The degradation half‐life (t_1/2_) of all BTM radicals is over 3500 times higher than that of TTM and PTM. The superior photostability of the D–A radicals benefits from the conjugation of acridine segments into the carbon‐centralized BTM radicals for providing steric shielding and unpaired electron delocalization performances. The highest decomposition temperature of these D–A radical was further determined to be 264, 256 , and 313 °C for DMA‐BTM, DPA‐BTM, and SFA‐BTM, respectively (Figure S12, Supporting Information). Furthermore, they maintained relatively good radical properties over 24 h incubation in boiling water, suggesting their superior thermal stability (Figure S13, Supporting Information). In addition, it is worth noting that we save the step of oxidation or reduction‐induced free radical generation compared to ionic radicals, which is also an important reason for obtaining superior photostability and thermal stability.^[^ ^10^ ^]^

**Figure 2:**
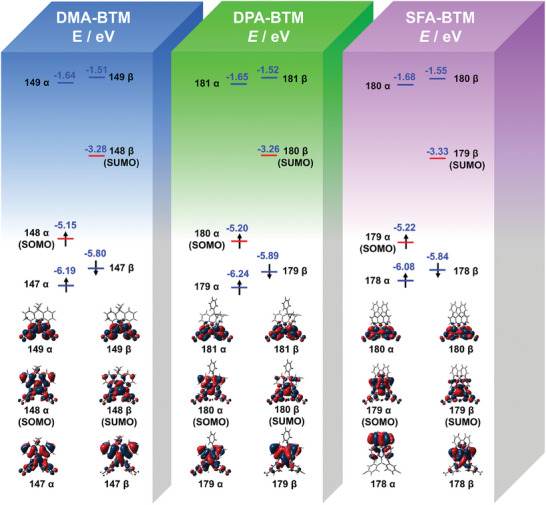
Quantum‐chemical analysis for the DMA‐BTM, DPA‐BTM, and SFA‐BTM radicals. The frontier molecular orbitals were calculated at B3LYP/6‐31+G(d,p) level of theory.

Frontier molecular orbitals of the D–A radicals were further analyzed using density functional theoretical (DFT) calculation. As illustrated in **Figure** **2**, DMA‐BTM, DPA‐BTM, and SFA‐BTM show similar distributions in frontier orbitals. As noted, the singly occupied molecular orbitals (SOMO, 148α for DMA‐BTM, 180α for DPA‐BTM, and 179α for SFA‐BTM) show an obvious extent toward the acridine groups, which means more delocalization of the unpaired electron that is beneficial to produce high stability of D–A radicals. The energy levels of SUMO of the D–A radicals do not change significantly and the energy levels of SOMO of DPA‐BTM and SFA‐BTM are slightly lower than that of DMA‐BTM. The cyclic voltammetry measurements further confirmed the tendency (Figure S14 and Tables S11 and S12, Supporting Information). The non‐radiative transition behaviors characterization of three‐D–A radical was further carried out by transient absorption spectroscopy. As demonstrated in Figure S15A–C (Supporting Information), transient absorption spectra reveal positive signals, which might be attributed to excited state absorption. The fitted lifetime of D–A radicals with exponential decay function is assigned to the picosecond span, implying that they are due to internal conversion (IC) to the ground state (Figure S15D–F, Supporting Information). In monoradical molecules, the non‐radiative transition rate is mostly determined by internal conversion efficiency.^[^ ^18^ ^]^ Thus, the return of the excited state to the ground state via non‐radiative relaxation is dominant. The IC coefficient from the lowest excited doublet state (D_1_) to the ground state (D_0_) of these D–A radicals was calculated by the Molecular Materials Property Prediction program.^[^ ^19^ ^]^ The IC coefficients of DMA‐BTM, DPA‐BTM, and SFA‐BTM are 6.0098 × 10^11^, 6.78 × 10^11^, and 1.4438 × 10^12^ S^−1^, respectively (Table S13, Supporting Information). As demonstrated, SFA‐BTM displays the most rapid IC efficiency, predicting the best non‐radiative transition among the three D–A radicals, which indicates that the SFA‐BTM molecule was preferred for further study (Figures S16 and S17, Supporting Information).

### 2.2 NPs Preparation and Photothermal Characterizations

**Figure 3:**
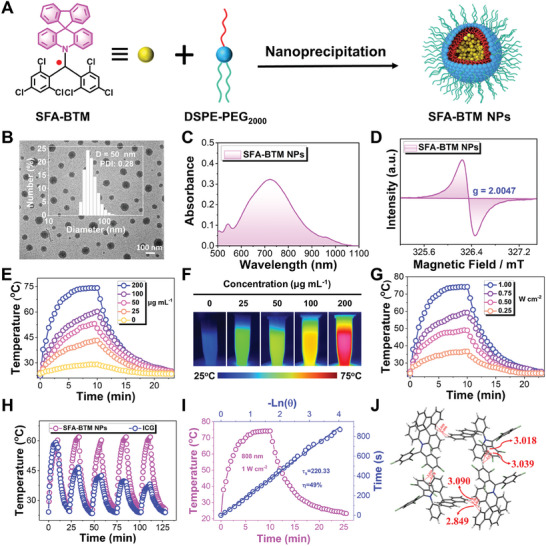
Characterizations and photothermal response of the SFA‐BTM NPs. A) Schematic illustration of the fabrication of SFA‐BTM NPs. B) The image of visualization of the morphology of SFA‐BTM NPs characterized by TEM, scale bar = 100 nm. Insert graph: size distribution of the SFA‐BTM NPs dispersed in water. C) UV–vis–NIR absorption spectrum of the SFA‐BTM NPs aqueous solution. D) EPR spectrum measured of SFA‐BTM NPs aqueous solution at 25 °C. The g value was calculated to be 2.0047. E) Temperature change pattern of the SFA‐BTM NPs aqueous solution upon 10 min irradiation of 808 nm laser (1 W cm^−2^) followed with natural cooling to ambient temperature. F) Infrared thermal images of the SFA‐BTM NPs dispersions at various concentrations. G) Time course of photothermal heating and cooling of the SFA‐BTM NPs aqueous solution (200 µg mL^−1^) at various power intensities of 808 nm laser. H) Photothermic resistance capabilities of the SFA‐BTM NPs and ICG upon 808 nm laser illumination (1 W cm^−2^) for five cycles (each cycle contains 10 min irradiation and 10 min cooling). I) Curves of photothermal heating and cooling until room temperature of SFA‐BTM NPs aqueous solution, and linear analysis of PCE according to the cooling process. J) Molecular packing of SFA‐BTM in crystallographic structure.

To improve water‐soluble performance, the resulting SFA‐BTM were fabricated into water‐dispersed nanoparticles (SFA‐BTM NPs) using amphiphilic polymer DSPE‐PEG_2000_ as carriers via a typical nanoprecipitation method as we previously reported (**Figure** **3**A).^[^ ^20^ ^]^ Transmission electron microscopy (TEM) characteristics indicated that the SFA‐BTM NPs had approximate spherical morphology with diameters of ≈30 nm (Figure 3B). The dynamic light scattering (DLS) measurement was employed to characterize the particle size of SFA‐BTM NPs, which measured to be ≈50 nm and kept stable over 1 week (Figure 3B; Figure S18, Supporting Information). In addition, DLS measurement also confirmed their stability in 10% FBS‐containing water and DMEM (Figure S19, Supporting Information). The zeta potential of ≈‐34.2 mV suggested the negative charge of SFA‐BTM NPs (Figure S20, Supporting Information). As illustrated in Figure 3C, the SFA‐BTM NPs have a broadened NIR absorption with a peak at 716 nm, which is consistent with SFA‐BTM in cyclohexane. The SFA‐BTM NPs also show emission at the NIR‐II region, which is consistent with the profile of its molecular state (Figure S21, Supporting Information). As noted by EPR spectroscopy, the SFA‐BTM NPs aqueous solution remains the radical property in accordance with the free SFA‐BTM (Figure 3D).

Next, the photothermal performances of SFA‐BTM NPs were investigated. As illustrated in Figure 3E–G, 808 nm laser irradiation produces a significantly increase in temperature of the SFA‐BTM NPs aqueous solution with the increasing irradiation time, while the water alone without NPs shows a slight temperature change in the same condition. Also, the temperature‐increasing phenomenon of SFA‐BTM NPs was proved to have concentration‐ and power‐dependent profiles. Infrared thermal images further confirmed the above‐noted photothermal performances (Figure 3F). The SFA‐BTM NPs showed good photothermic resistance capabilities after 5 heating‐cooling cycles, while the FDA‐approved indocyanine green (ICG) was almost lost its photothermal performance under the same photothermal activated conditions (808 nm laser, 1 W cm^−2^) (Figure 3H). The absorption spectra measurements before and after 808 nm laser irradiation confirmed the good photostability of SFA‐BTM NPs (Figure S22, Supporting Information). After 6 days of incubation in 10% FBS‐containing water and DMEM, the photothermal properties of SFA‐BTM NPs remained stable (Figure S23, Supporting Information). SFA‐BTM NPs show a comparable PCE (49%) to that of the recently reported PTAs (Figure 3I; Table S14, Supporting Information),^[^ ^15a,c^ ^]^ which was calculated according to a reported literature.^[^ ^6c^ ^]^ As indicated by single crystal analysis, the high photothermal conversion property of SFA‐BTM NPs would be mainly rooted from the anisotropic stacking in the aggregation state that benefits heat production (Figure 3J). The assumption was further demonstrated by the calculation of the reorganization energies at the crystal phase. As demonstrated, SFA‐BTM presents the highest excited state relaxation energy (0.320 eV) in the crystal phase, compared with 0.257 eV for DMA‐BTM and 0.319 eV for DPA‐BTM, which implies efficient internal conversion efficiency is achieved for photothermal conversion in aggregation state (Figure S24, Supporting Information). These results suggest that radical SFA‐BTM NPs have been excellent PTAs with good stability.

### 2.3 In Vitro Photothermal Ablation

**Figure 4:**
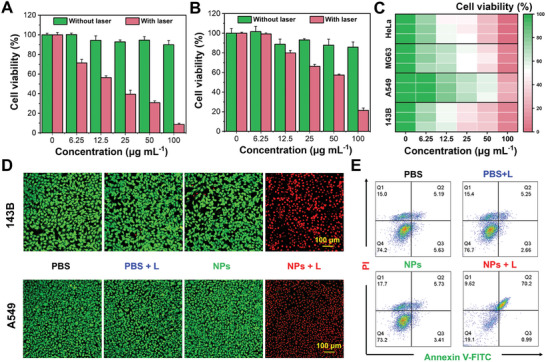
In vitro photothermal therapy. Cell survivals of A)143B cells, B) A549 cells, and other tumor cells C) after photothermal therapy (1 W cm^−2^ power density of 808 nm laser irradiation for 5 min) using various concentrations of SFA‐BTM NPs. Data were presented as mean ± standard deviation, which was calculated from the data of three biologically independent samples. D) Cell imaging of 143B and A549 cells after various treatments. Green‐fluorescent cells show the alive, whereas red‐fluorescent cells show the dead. Scale bar = 100 µm. E) Apoptosis analysis of various treated 143B cells by flow cytometry.

Motivated by the good photothermal performances, the in vitro antitumor activity evaluation of SFA‐BTM NPs was conducted by methylthiazolyl tetrazolium (MTT) assay upon the irradiation of 808 nm laser. As shown in **Figure** **4**A, osteosarcoma cells 143B treated with SFA‐BTM NPs alone at various concentrations have a small or even a negligible effect on cell viability, suggesting the good biocompatibility of SFA‐BTM NPs. However, SFA‐BTM NPs show concentration‐dependent killing capability toward 143B cells upon the laser irradiation of 808 nm. Notably, ≈90% of the tumor cells were eradicated after 5 min of laser irradiation at a concentration of 100 µg mL^−1^ of SFA‐BTM NPs. The similar photoablation capability was further confirmed in human lung cancer cells A549, cervical cancer cells HeLa, and osteosarcoma cell line MG‐63, suggesting broad‐spectrum activity of SFA‐BTM NPs against cancer cells (Figure 4B,C). Moreover, the preliminary biosafety evaluation of SFA‐BTM NPs toward normal cells NIH‐3T3 was performed and the results show good biosafety even the concentration is increased to 100 µg mL^−1^ (Figure S25, Supporting Information).

In addition, the live/dead cell staining was employed to further indicate the in vitro photoablation performance of SFA‐BTM NPs under the imaging of a confocal microscope. As shown in Figure 4D, treatment with SFA‐BTM NPs or 808 nm laser irradiation alone shows that almost all the 143B cells were completely stained with green fluorescence, indicating good cell survival without any killing. However, upon the 808 nm laser irradiation, the SFA‐BTM NPs induced the tumor cells to appear red fluorescence, which indicates that the tumor cells are eliminated upon the photothermal ablation of SFA‐BTM NPs. The consistent results were obtained in live/dead cell staining on A549. In addition, the photothermal‐induced apoptosis of 143B cells was evaluated via flow cytometry analysis, and found that SFA‐BTM NPs followed by 808 nm laser irradiation caused obvious apoptosis (Figure 4E). These results demonstrate the good in vitro photoablation effect of SFA‐BTM NPs toward tumor cells.

### 2.4 In Vivo Multimodal Bioimaging

**Figure 5:**
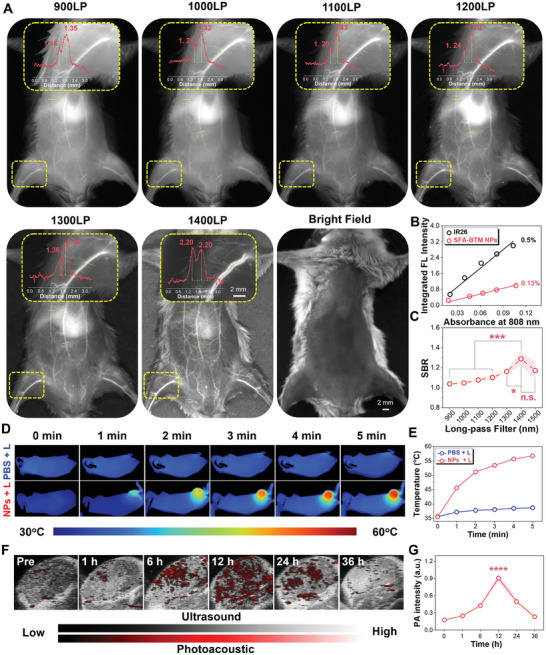
In vivo bioimaging using SFA‐BTM NPs. A) Whole‐body NIR‐II bioimaging with sequential LP filters from 1000 to 1400 nm. Inset: NIR bioimaging and SBR analysis of the labeled vessel under different LP filters. B) Quantum yield analysis of IR26 in DCE and SFA‐BTM NPs in water under 808 nm excitation. C) SBR analysis of whole‐body blood vessels labeled in (A). D) Photothermal images of 143B tumor‐bearing mice in different treatment groups (SFA‐BTM NPs: 10 mg kg^−1^) under 808 nm laser irradiation (1 W cm^−2^). E) Temperature changes of tumor location at different time intervals. F) Photoacoustic images of tumor location at the post‐intravenous injection of SFA‐BTM NPs. G) Quantitative PA intensities of tumor locations from (F) plotted against the time. The excitation wavelength used in photoacoustic imaging was 808 nm. Data are presented as mean ± standard deviation based on three biologically independent mice. The statistical difference was calculated using one‐way ANOVA with Tukey's test (^*^ *P* < 0.05, ^**^ *P* < 0.01, ^***^ *P* < 0.005, ^****^ *P* < 0.001). n.s. indicates no significance.

Fluorescence angiography is of great importance for disease diagnosis. As anticipated, SFA‐BTM NPs' main emission is found in the NIR‐II region, indicating that they are capable of performing in vivo NIR‐II fluorescence angiography. Thus, the whole‐body fluorescence angiography of SFA‐BTM NPs was assessed in mice upon the 808 nm excitation. As shown in **Figures** **5**A and S26 (Supporting Information), the NIR‐II fluorescence signal with a range of long‐pass (LP) filters (from 900 to 1500 nm) clearly delineates the vascular architecture with numerous tiny capillaries branching from larger vessels in the abdomen. Using the commercially available IR26 (0.5% in 1,2‐dichloroethane) as a standard, the quantum yield (QY) of SFA‐BTM NPs aqueous solution was determined to be 0.13% (Figure 5B). Notably, the NIR‐II images at the 1400 nm LP filter have the higher signal‐to‐background ratio (SBR, 1.29) than the images using the other LP filters, indicating good resolution (Figure 5C).^[^ ^21^ ^]^ Also, SBR analysis of the blood vessels of the hind limbs in mice found that the NIR‐II imaging at the 1400LP filter exhibited biggest SBR of 2.20. Taken together, SFA‐BTM NPs have high‐resolution NIR‐II angiography performance, offering benefits for in vivo imaging.

Encouraged by the high photothermal conversion efficiency of SFA‐BTM NPs, in vivo tumor photothermal and photoacoustic (PA) imaging analyses were further performed to identify the detailed photothermal effect and tumor distribution in the live animals. The mice were intravenously injected with SFA‐BTM NPs and then exposed to an 808 nm laser. The obvious rise in temperature at tumor sites was visualized by infrared thermal imaging and showed a gradual increase over time (Figures 5D,E). In contrast, the mice without SFA‐BTM NPs treatment only display a slight temperature rise in tumor locations under the same condition. These results validate the ability of SFA‐BTM NPs for photothermal imaging by triggering the temperature increase of tumor upon 808 nm laser irradiation. Next, in vivo photoacoustic (PA) imaging was conducted to evaluate the photoacoustic potential and further confirm the tumor‐specific of SFA‐BTM NPs. As illustrated in Figure 5F,G, after intravenous injection of SFA‐BTM NPs for 1 h, photoacoustic signals began to appear in tumor locations and gradually strengthened over time. After achieving the maximum intensity in the tumor at 12 h post‐injection, PA signals displayed gradual recession and were almost completely quenched at 36 h post‐injection. We further studied tumor‐targeting properties of SFA‐BTM NPs by high‐performance liquid chromatography‐tandem mass spectrometry (HPLC/MS) and found the accumulation of SFA‐BTM NPs in tumor tissue peaked at 12 h post‐injection (Figure S27, Supporting Information), which is consistent with the PA imaging results. In summary, our findings validate that SFA‐BTM NPs are adaptable radical materials with promise for angiography and tumor imaging.

### 2.5 In Vivo Treatment and Biosafety Evaluation

**Figure 6:**
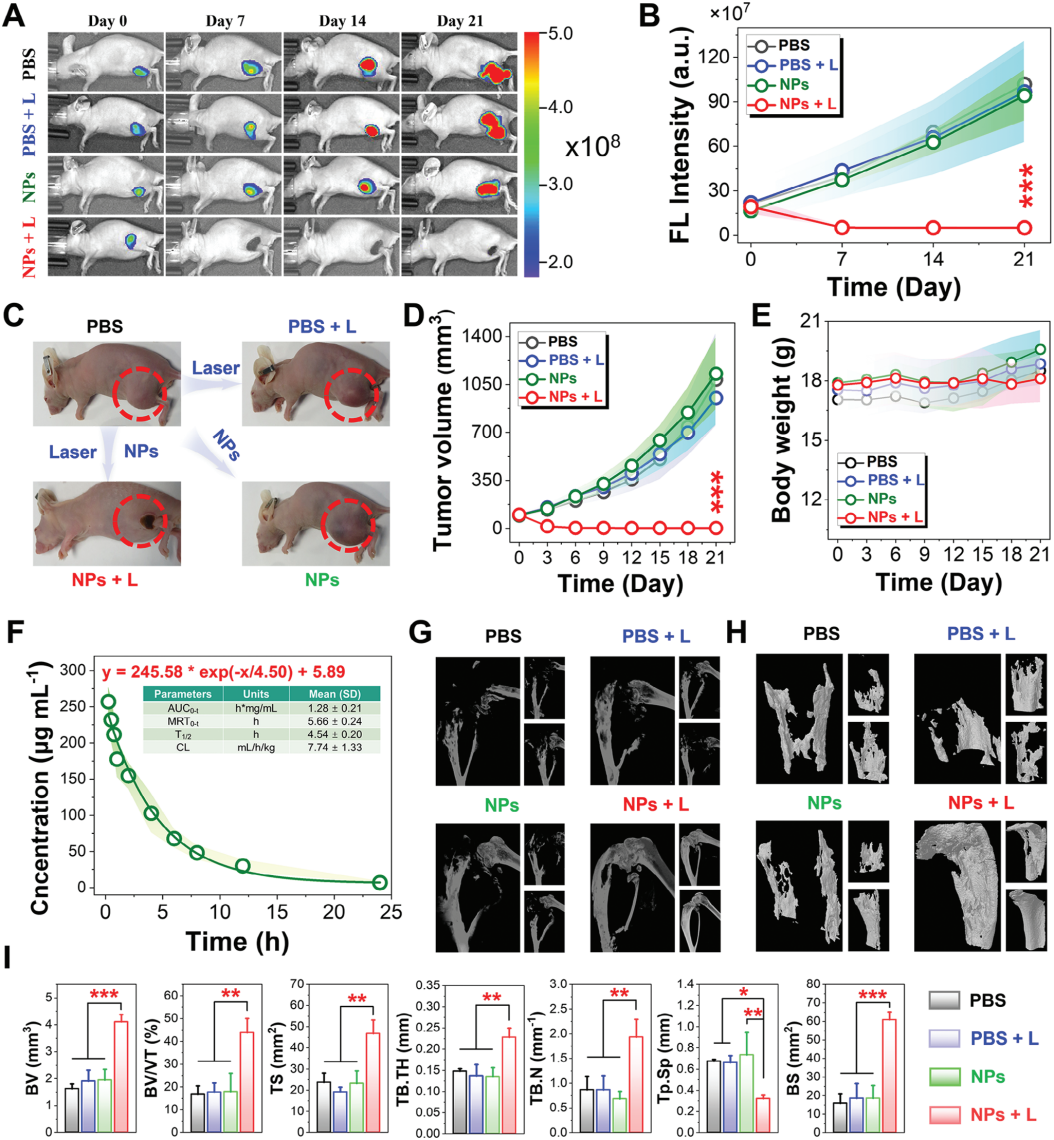
In vivo photothermal ablation and bone repair. A) Fluorescence visualization of the treatment effect of PTT on 143B tumor‐bearing mice. B) Quantitative analysis of fluorescence intensities at tumor sites. C) Comparative photographs of 143B tumor‐bearing mice after various treatments. D) Tumor volume curves of the mice in various treatments. E) Body‐weight changes of the mice in various treatments. F) In vivo pharmacokinetics of SFA‐BTM NPs after intravenous administration. AUC area under the curve, MRT mean residence time, T_1/2_ terminal half‐life, CL clearance. G) 3D reconstruction in micro‐CT images of tibias from three treated mice. H) 3D sagittal reconstruction of tibia fragments from each mouse in the same treatment group. I) Plots of the parameters of bone architecture with various treatments. BV bone volume, BV/TV bone volume/total volume, TS tibia space, Tb.Th trabecular thickness, Tb.N trabecular numbers, Tb.Sp trabecular separation, BS bone surface. Data from five (B, D, E), four (F) and three (I) biologically independent mice were presented as mean ± standard deviation. The statistical difference was calculated using one‐way ANOVA with Tukey's test (^*^ *P* < 0.05, ^**^ *P* < 0.01, ^***^ *P* < 0.001).

The in vivo photothermal ablation of SFA‐BTM NPs was further investigated on the mice xenografted 143B‐RFP tumor in situ that inherently expressed red fluorescent protein (RFP) for visualizing the tumor. As illustrated by monitoring the intensity of red fluorescence in **Figure** **6**A,B, mice treated with SFA‐BTM NPs plus laser irradiation exhibit a rapid decline in fluorescence intensity at tumor sites and completely disappear at the 7‐day post‐treatment without any recurrence in the 21‐day treatment. In contrast, SFA‐BTM NPs alone and PBS plus laser irradiation have no impact on tumor growth in mice. The actual measurements of tumor volume by an electronic digital calliper every other day further confirmed that the SFA‐BTM NPs treatment with laser irradiation could efficiently eliminate the tumor without any relapse during the 21‐day observation (Figure 6C,D; Figures S28 and S29, Supporting Information). Tumor biopsies of the treated mice by hematoxylin and eosin (H&E) staining were conducted and showed that SFA‐BTM NPs treatment plus laser irradiation can induce typical morphological features of apoptotic cells in tumor tissue while other groups have not shown any apoptosis (Figure S30, Supporting Information). These results further demonstrate the good anti‐tumor performances of SFA‐BTM NPs via photoablative action.

It should be noted that bone tumor generally causes serious osteoclastic resorption of the bone closest to tumor, which may result in a range of tough complications and further increase the mortality rate. Thus, the efficient suppression of osteoclastic bone resorption is crucial to the treatment of bone tumor. Motivated by the good elimination of in situ bone tumor, we next detailed investigated the bone erosion inhibition properties of the mice with SFA‐BTM NPs treatment. The micro‐CT (also called micro‐computed tomography) reconstruction of nearby bone was illustrated in Figure 6G, and the results show that the mice with SFA‐BTM NPs treatment plus laser irradiation have intact morphology of nearby bone without a minimal erosive lesion while the other three treatments could not inhibit the osteoclastic bone resorption. The details provided by 3D models of the tibial transverse sections and profiles further confirm that SFA‐BTM NPs treatment plus laser irradiation could efficiently inhibit osteoclastic bone resorption and favor bone repairing (Figure 6H; Figure S31, Supporting Information). Moreover, the key indicators of bone architecture, mainly include bone volume (BV), bone volume/total volume (BV/TV), tibia space (TS), trabecular thickness (Tb.Th), trabecular numbers (Tb. N), trabecular separation (Tb. Sp), and bone surface (BS) were quantitatively analyzed and summarized in Figure 6I. Upon SFA‐BTM NPs treatment plus laser irradiation, the indicators including BV, BV/TV, TS, Tb.Th, Tb. N, and bone BS of the treated mice display significantly increasing while the parameter of Tb. Sp is greatly decreased, indicating the good suppression of the nearby bone destruction by SFA‐BTM NPs treatment plus laser irradiation.

For biosafety evaluations, we first evaluated the potential toxicity of the SFA‐BTM NPs toward enzymes. Four enzymes, lipase, acetylcholinesterase, catalase, and superoxide dismutase were employed in this evaluation and found that SFA‐BTM NPs hardly affected the activity of these enzymes (Figure S32, Supporting Information). Then the biocompatibility of 1 W cm^−2^ 808 nm laser was also demonstrated by H&E staining of the irradiated skin, and no laser injury to the skin was observed after 5 min of irradiation (Figure S33, Supporting Information). Furthermore, we measured the body weight change of the mice after various treatments, and no noticeable difference in body weight was observed between the control and treatment groups (Figure 6E). The tissue biopsy and H&E staining of main organs such as heart, liver, spleen, lung, and kidney extracted from the treated mice after 21‐day treatment was carried out (Figure S30, Supporting Information). There were no apparent abnormalities or damage toward organs after treatments. The pharmacokinetic performance of SFA‐BTM NPs after intravenous administration was further assessed by determination of concentration in blood at various times via HPLC/MS, and the results indicated that the half‐life (t_1/2_) of SFA‐BTM NPs was calculated to be 4.5 h, indicating a long blood circulation time (Figure 6F). Furthermore, the blood routine examinations and blood biochemistry analysis were performed at the end of various treatments. The results show that the SFA‐BTM NPs treatment plus laser irradiation present a negligible change to the noted biochemical parameters (Figure S34, Supporting Information). Hemolysis test further demonstrated good blood biocompatibility of SFA‐BTM NPs (Figure S35, Supporting Information). These findings demonstrate that SFA‐BTM NPs have relatively high biosafety for in vivo cancer treatment.

## 3 Conclusion

In this work, we designed and synthesized various D–A radicals and fabricated SFA‐BTM NPs as effective photothermal materials. The D–A radicals show a large extinction coefficient at the NIR‐I region and tails extended to 1000 nm with obvious NIR‐II emission, and outstanding photostability and thermostability. Owing to the rapid doublet internal conversion, radical molecule SFA‐BTM is expected to have the best non‐radiative transition, and the NPs have been shown to exhibit excellent performance for photoacoustic and photothermal applications, with a comparable PCE of 49%. Due to their efficient NIR‐II emission, the SFA‐BTM NPs achieved high‐resolution whole‐body angiography by the NIR‐II bioimaging upon 808 nm excitation. Meanwhile, by high stability and biosafety, SFA‐BTM NPs achieve complete elimination of cancer cells in vitro, and are successfully carried out on 143B tumor‐xenograft mice with excellent tumor ablation and outstanding curative effect on bone erosion under irradiation of 808 nm laser. In conclusion, this work provides a new insight into D–A radical materials as NIR‐II emissive theranostic agents for the development of the innovative theranostic platforms.

## 4 Experimental Section

### Ethical Statement

All animal experiments were carried out following national and international regulations regarding animal experimentation and the protocols had been approved by the Animal Care and Use Committee of Shenzhen Institute of Advanced Technology (SIAT‐IACUC‐20210826‐YGS‐ZHZX‐ZW‐A1109‐01) and the Institutional Ethical Committee of Animal Experimentation of Soochow University (SUDA20241210A03).

### Statistical Analysis

Origin Pro 2021 and Microsoft Office Excel (Office 365) were used for statistical analysis. ImageJ was used for the image analysis. Flow Jo V10 was used to analyze data collected on the flow cytometer. The sample sizes in statistical analysis were n = 3 or 5 unless otherwise noted, while the data were shown as mean ± standard deviation. Probability (*P*)‐values were calculated by one‐way analysis of variance (ANOVA) with Tukey's test, *P* < 0.05 was considered significant and n. s. represent no significance.

## Conflict of Interest

The authors declare no conflict of interest.

## Supporting information



Supporting Information

## Data Availability

The data that support the findings of this study are available from the corresponding author upon reasonable request.
