# Supplementary material for: NIR‐II Emissive Persistent Neutral π‐Radical with Rapid Doublet Internal Conversion for Efficient Cancer Photothermal Theranostics
Source: Adv Sci (Weinh). 2025 Feb 25;12(15):2411733. doi: 10.1002/advs.202411733 (PMC12005734; doi:10.1002/advs.202411733)
Supplement: Supplementary file 1 — Supporting Information [file ADVS-12-2411733-s001.docx]

Supporting Information

**NIR-II Emissive** **Persistent Neutral π-Radical with Rapid** **Doublet Internal Conversion for Efficient Cancer Photothermal Theranostics**

*Qi Zhao, Chunxiao Wu, Yijian Gao, Jing Long, Wei Zhang,* Yanan Chen, Yuliang Yang, Yu Luo, Yuxiao Lai, Houyu Zhang, Xiankai Chen, Feng Li,* Shengliang Li**

**Content**

1. **Experimental Procedures**

**Materials and measurements**

**Synthesis and characterization of radicals**

**Stability evaluation of radical properties**

**Computational methods**

**Preparation and characteristics of radical nanoparticles**

Preparation procedures of radical nanoparticles

Physicochemical characteristics and measurements

Stability evaluation of nanoparticles

Photothermal properties

Calculation of photothermal conversion efficiency

**Cell experiments**

Cytotoxicity and biocompatible evaluations

Live/dead cell staining

Flow cytometer analysis

**Animal experiments**

Whole-body NIR-II fluorescence imaging

Establishment of tumor model

*In vivo* tumor imaging

Photothermal tumor ablation

Pharmacokinetics study

*In vivo* biodistribution

**Biosafety Evaluation**

*In vivo* biosafety

Biosafety evaluation of laser irradiation

Hemolysis test

Enzyme activity evaluation

1. **Supplementary Figures**

**Figures S1 to S33**

1. **Supplementary Tables**

**Tables S1 to S14**

1. **References**

1. **Experimental Procedures**

**Materials and measurements**

All raw materials and solvents were commercially available. ^1^H NMR spectra were recorded on a Bruker AVANCEIII500 NMR spectrometer with tetramethylsilane (TMS) as the internal standard. GC-MS mass spectra were recorded on a Thermo Fisher ITQ1100 mass spectrometer. High-resolution liquid chromatography (HRLC) mass spectra were recorded on a Bruker Agilent1290-micrOTOF Q II mass spectrometer. MALDI-TOF mass spectra were recorded on a Bruker Autoflex speed TOF/TOF mass spectrometer. EPR spectra were recorded on Bruker ELEXSYS-II E500 CW-EPR spectrometer and JES-X320 spectrometer at 298K. Transient absorption spectra were collected on a HELIOS Fire femtosecond transient absorption spectrometer. For DMA-BTM and DPA-BTM, their crystals were obtained at room temperature and the results of single crystal X-ray diffraction were obtained from a Bruker Apex II CCD diffractometer at 295K, with graphite-monochromated MoKa radiation (λ=0.71073 Å). For SFA-BTM, its crystal was obtained at -35^o^C and its X-ray diffraction was measured at 100 K. Thermal stability measurements were carried out on a TAINSTRUMENTS Q500 TGA analyser. The cyclic voltammetry (CV) measurements were performed using a CH Instruments CHI660E electrochemical analyser with a glass carbon disk as the working electrode, a platinum wire as the counter electrode, and Ag/Ag^+^ as the reference electrode under a sweeping rate of 50 or 100 mV s^-1^. And the ferrocenium/ferrocene redox couple was used as an internal standard. Biodistribution and pharmacokinetics were studied *via* high-performance liquid chromatography (UltiMate 3000 RS)-tandem mass spectrometry (TSQ Quantum).

**Synthesis and characterization of radicals**

**Synthesis of DMA.** The specific procedure to synthesize DMA was totally followed a mature method reported before.^[1]^ GC-MS (m/z): [M^+^] calculated for C_15_H_15_N, 209.12; found 209.06. ^1^H NMR (500 MHz, DMSO-*d6*) δ 8.82 (s, 1H), 7.34 (d, J = 7.2 Hz, 2H), 7.05 (td, J = 7.7, 1.3 Hz, 2H), 6.83 - 6.77 (m, 4H), 1.49 (s, 6H).

**Synthesis of HBTM-Br.** HBTM-Br was synthesized follow the same method reported by our group recently.^[2]^

**Synthesis of DMA-BTM.** Under argon atmosphere, sodium hydride (60% in oil, 0.8 g, 20 mmol) was dispersed in a solution of anhydrous dimethyl sulfoxide (90 mL), then DMA (2.1 g, 10 mmol) dissolved in anhydrous dimethyl sulfoxide (10 mL) was added dropwise and stirred until no gas was generated in the reaction system. After stirring for half an hour, HBTM-Br (2.3 g, 5 mmol) was added into the reaction mixture directly without dissolved in the solvent. Then the reaction system was stirred overnight at 55^o^C. Afterwards, the mixture was cooled to room temperature and poured into saturated ammonium chloride solution (200 mL). Lately, the mixture was extracted with CH_2_Cl_2_ (100 mL×3). The organic layer was combined and dried over anhydrous magnesium sulphate. After removing the solvent under reduced pressure, the residue was purified by flash column chromatography (silica gel, dichloromethane/petroleum ether = 1:15). DMA-BTM was obtained as atrovirens solid in 6% (0.18 g) yield. HRLC-MS (*m/z*): [M^+^] calcd for C_28_H_18_C_l6_N, 579.9541; found, 579.9571.

**Synthesis of DPA.** DPA was synthesized from a mature method reported before.^[3]^ GC-MS (m/z): [M^+^] calculated for C_25_H_19_N, 333.15; found 333.03. ^1^H NMR (500 MHz, DMSO-*d6*) δ 9.08 (s, 1H), 7.26 (t, *J* = 7.4 Hz, 4H), 7.21 (t, *J* = 7.1 Hz, 2H), 7.15 (t, *J* = 7.5 Hz, 2H), 6.93 (d, *J* = 7.8 Hz, 2H), 6.85 (d, *J* = 7.4 Hz, 4H), 6.81 (t, *J* = 7.5 Hz, 2H), 6.65 (d, *J* = 7.7 Hz, 2H).

**Synthesis of DPA-BTM.** Under argon atmosphere, sodium hydride (60% in oil, 132 mg, 3.3 mmol) was dispersed in a solution of anhydrous dimethyl sulfoxide (15 mL), then DPA (550 mg, 1.65 mmol) dissolved in anhydrous dimethyl sulfoxide (10 mL) was added dropwise and stirred until no gas was generated in the reaction system. After stirring for half an hour, HBTM-Br (500 mg, 1.1 mmol) was added into the reaction mixture directly without dissolved in the solvent. Then the reaction system was stirred overnight at 55^o^C. Afterwards, the mixture was cooled to room temperature and poured into saturated ammonium chloride solution (100 mL). The resulting precipitate was filtered and dissolved with dichloromethane. After removing the solvent under reduced pressure, the residue was purified by flash column chromatography (silica gel, dichloromethane/petroleum ether = 1:4). DPA-BTM was obtained as atrovirens solid in 13% (100 mg) yield. MALDI-TOF (*m/z*): [M^+^] calcd for C_38_H_22_C_l6_N, 703.9854; found, 703.8265.

**Synthesis of SFA-BTM.** Under argon atmosphere, sodium hydride (60% in oil, 132 mg, 3.3 mmol) was dispersed in a solution of anhydrous dimethyl sulfoxide (15 mL), then commercially available SFA (547 mg, 1.65 mmol) dissolved in anhydrous dimethyl sulfoxide (10 mL) was added dropwise and stirred until no gas was generated in the reaction system. After stirring for half an hour, HBTM-Br (500 mg, 1.1 mmol) was added into the reaction mixture directly without dissolved in the solvent. Then the reaction system was stirred overnight at 55^o^C. Afterwards, the mixture was cooled to room temperature and poured into saturated ammonium chloride solution (100 mL). The resulting precipitate was filtered and dissolved with dichloromethane. After removing the solvent under reduced pressure, the residue was purified by flash column chromatography (silica gel, dichloromethane/petroleum ether = 1:4). SFA-BTM was obtained as atrovirens solid in 3% (20 mg) yield. MALDI-TOF (*m/z*): [M^+^] calcd for C_38_H_20_C_l6_N, 701.9697; found, 701.4252.

**Stability evaluation of radical properties**

The photostability of three D-A radical molecules was evaluated under 365-nm UV irradiation (power density 1.3 W cm^-2^). The radicals were diluted by toluene to 100 μM and transfer 200 μL of each sample to an EPR tube. The radical properties were measured on EPR spectrometer before and after 70 min irradiation. The thermal stability was assessed by measuring the change in EPR signal intensity before and after incubation in boiling water. DMA-BTM NPs and DPA-BTM NPs were fabricated by the above-mentioned nanoprecipitation method. Transfer 500 μL of nanoparticle solutions (2 mg mL^-1^) to tubes, immerse in boiling water and keep warm for 24 h. The nanoparticle solutions incubated at room temperature served as control. Then the resulting samples were encapsulated into capillaries and their radical properties were measured on EPR spectrometer.

**Computational methods**

Theoretical calculations based on DFT and TD-DFT methods were performed on Gaussion09 series of programs using the B3LYP function and 6-31+G(d,p) basis. The reorganization energies were calculated using ONIOM(B3LYP/6-31+G(d,p) :UFF)-EE model. Cyclohexane as a solvent was taken into consideration in all calculations.

The internal conversion rate constant of three radicals were calculated by MOlecular MAterials Property Prediction Package (MOMAP) which was developed by Zhigang Shuai’s group.^4^ The non-radiative decay internal rate constant was described by the following equation according to their previous work:

$$k_{ic}=\sum_{kl} \frac{1}{\hbar^{2}}R_{kl}\int_{-\infty}^{\infty} ⅆte^{i\omega_{if}t}Z_{i}^{-1}\rho_{ic,kl}$$

Where $\hbar$ is the planck constant, R_kl_ is the nonadiabatic electronic coupling, ω_if_ is the electronic transition energy, Z_i_ is the partition function and $\rho_{ic,kl}$ is the thermal vibration correlation function. The detailed expression of $\rho_{ic,kl}$ can be found in the reference.^[4]^

The geometries of radicals in ground and excited state were optimized using B3LYP function and 6-31G(d,p) basis with Gaussian 09. Frequency calculations were carried out to ensure that the optimized geometries were minima on the potential energy surface, in which no imaginary frequencies were observed. The non-adiabatic coupling matrix element calculations used the same function and basis as geometry optimization.

**Preparation and characteristics of radical nanoparticles**

Preparation of radical nanoparticles

To obtain water-solubility, the radical molecules were co-assembled with amphipathic polymer DSPE-PEG_2000_ to create nanoparticles using a conventional nanoprecipitation method. Detailly, 0.5 mg mL^-1^ of SFA-BTM and 5 mg mL^-1^ DSPE-PEG_2000_ solutions were prepared by dissolving in THF solvent, and then mixed them well in a one-to-one volume ratio. The mixture was slowly dropped into 9 ml of deionized water to form nanoparticles with vigorously stirred. After removing the remaining THF with a rotary evaporator, the resulting SFA-BTM NPs were filtered with a 0.2 μm membrane and concentrated *via* ultrafiltration (Millipore, size of 100 kDa). The prepared NP solution was kept in 4 °C for further use.

Physicochemical characteristics and measurements

The absorption spectra of NPs were collected on a UV-vis-NIR scanning spectrophotometer (Shimadzu UV-2550). The dynamic light scattering (DLS) measurement was employed to analyse the size distribution of NPs at room temperature using a Malvern Zetasizer Nano ZS size analyser. Transmission electron microscopy (TEM) was used to visualize the morphological features of nanoparticles.

Stability evaluation of nanoparticles

The size stability and photothermal performance of SFA-BTM NPs were evaluated in the physiological environment. The dispersion stability of SFA-BTM NPs was assessed by analysing the changes in size and polydispersity index (PDI) over 8 days by dynamic light scattering. The concentrated SFA-BTM NPs were diluted with water, water + 10% fetal bovine serum (FBS) and DMEM + 10% FBS to a concentration of 100 μg mL^-1^. Then the changes on size and photothermal performance were monitored by DLS and infrared thermal imager every other day.

Photothermal properties

500 μL solution of SFA-BTM NPs with various concentrations (0, 25, 50, 100, and 200 μg mL^-1^) were prepared and then exposed to 808 nm laser at different power densities (0.25, 0.5, 0.75, and 1 W cm^-2^), and pure water as the control. The photothermal conversion performances were studied by monitoring the temperature rise caused by the NIR laser acquired by a high-accurate thermal imaging system. Repeated laser irradiation was conducted to reveal the stability of SFA-BTM NPs towards heat. Firstly, the 100 μg ml^-1^ SFA-BTM NPs solution (0.5 mL) was prepared and exposed to 808 nm laser. ICG (100 μg ml^-1^) was employed as a control. The power density of 808 nm laser was fixed at 1 W cm^-2^. Then the laser was switched off after 10 min irradiation, and SFA-BTM NPs solution was cooled to room temperature. During this time, temperature changes were recorded every 30 seconds. Five cycles were tracked in all.

Calculation of photothermal conversion efficiency

200 μg mL^-1^ SFA-BTM NPs solution was prepared in a 0.5 mL tube and 808 nm laser was employed to excite the photothermal effect with a fixed power density at 1 W cm^-2^. The changes of temperature during heating and cooling processes of SFA-BTM NPs solution were recorded continuously. The PCE (η) can be determined by the following equations according to the previous report:^[5]^

|  | $\boldsymbol{\eta=}\frac{\boldsymbol{hS}\left( \boldsymbol{T}_{\boldsymbol{max}}\boldsymbol{-}\boldsymbol{T}_{\boldsymbol{surr}} \right)\boldsymbol{-}\boldsymbol{Q}_{\boldsymbol{dis}}}{\boldsymbol{I}\left( \boldsymbol{1-}\boldsymbol{10}^{\boldsymbol{-}\boldsymbol{A}_{\boldsymbol{808}}} \right)}$ | **(1)** |
| --- | --- | --- |
|  | $\boldsymbol{hS}\mathbf{=}\frac{{\boldsymbol{C}_{\boldsymbol{D}}\boldsymbol{m}}_{\boldsymbol{D}}}{\boldsymbol{\tau}_{\boldsymbol{s}}}$ | **(2)** |

**Cell experiments**

Cytotoxicity and biocompatible evaluations

143B, Hela and A549 cells (5×10^3^ per well) were seeded in 96-well plates and grown overnight, then exposed to varied concentrations (0, 6.25, 12.5, 25, 50 and 100 μg ml^-1^) of SFA-BTM NPs for 4 h. Three replicate wells were preset for each concentration. The photothermal treatment was performed by irradiated cells with an 808 nm laser with a power density of 1 W cm^-2^ for 5 min. After 24 h culturation, the treated cells and 0.5 mg mL^-1^ MTT were co-incubated for 4 h and DMSO was applied to replace medium to dissolve formazan. The cytotoxicity was assessed by recording optical densities (OD) at 570 nm. The *in vitro* biocompatible of SFA-BTM NPs was performed on normal cells (NIH-3T3) using MTT assay without irradiation.

Live/dead cell staining

143B and A549 cells were pre-cultured in confocal dishes (35 mm) for 24h. After co-incubation with SFA-BTM NPs (100 μg mL^-1^) for 4 h, cells were irradiated with 808 nm laser (1 W cm^-2^, 5 min) and no treated cells as control. The treated cells were cultured overnight and stained by the Calcein AM/Propidium iodide Cell Viability/Cytotoxicity Assay Kit (Beyotime Biotech) according to the manufacturer’s instructions, followed by fluorescent imaging on an infrared laser equipped confocal laser scanning microscope (Leica SP5).

Flow cytometer analysis

143B cells were collected and grew in a 12-well plate at a cell density of 1 × 10^5^. After overnight incubation, 143B cells were exposed to 100 μg mL^-1^ SFA-BTM NPs for 4 h, then an 808 nm laser at a power density of 1 W cm^-2^ was applied to afford 5 min of photothermal therapy. Discarding SFA-BTM NPs, 143B cells were cultured in DMEM for 24 h. The resulting 143B cells were harvested and stained with Annexin V-FITC/PI kit (Beyotime Biotech), then 10000 cells of each sample were analysed by flow cytometer for apoptosis evaluation.

**Animal experiments**

Whole-body NIR-II fluorescence imaging

Every Balb/c mouse used in the experiment weighed less than 20 g. Anesthesia was applied to the Balb/c mice prior to imaging. The mice got an intravenous injection of SFA-BTM NPs (2.50 mg mL^-1^, 100 µL). Following the 808 nm laser excitation of the mice, NIR-II fluorescence images were captured using a NIRvana camera equipped with several long-pass filters (900-1500 nm).

Establishment of tumor model

BALB/c female nude mice (approximately 20 g, 5 weeks old), purchased from Beijing Vital River Company (Beijing, China), were chosen to establish the orthotopic bone-tumor model. After injection of cavum medullare of tibias with 50 μL 143B-RFP cells solution (5×10^5^ cells in PBS), the development of bone tumor was tracked by employing an animal imaging system (IVIS, Lumina-II, Caliper Life Sciences). Mice displaying red fluorescence at the tibia lesion were chosen for *in vivo* treatments.

*In vivo* tumor imaging

143B tumor-bearing mice with a uniformed tumor volume of 100 mm^3^ were selected and randomized into groups A and B. Group A was the treatment group and SFA-BTM NPs (10 mg kg^-1^) were given intravenously, while mice in group B received an equal volume of PBS as a control group. At 12 h post-injection, mice were anaesthetized and the photothermal imaging of SFA-BTM NPs was initiated by 808 laser (1 W cm^-2^). The Fluke thermal imaging camera (Ti400) was employed to record images of the temperature change at the tumor sites every minute over five minutes. Three tumor-bearing mice were subjected to PA imaging by using a Vevo LAZR-X system. The tumor was covered with ultrasonic coupling gel. PA imaging was conducted before and after intravenous injection of SFA-BTM NPs solution (final SFA-BTM NPs content: 10 mg kg^-1^). The system setting parameters were as follows: frequency: 40 MHz, wavelength range: 680-970 nm, PA gain: 40 dB, gain:13 dB, depth:13.00 nm, width: 10.08 mm, PA acquisition: single, and wavelength: 808 nm.

Photothermal tumor ablation

Female BALB/c nude mice xenografted with 143B-RFP tumor showed red fluorescence at tumor sites during imaging, the antitumor efficiency can be measured by changes in the intensity of the red fluorescence. The animal number in each group was 5, which was calculated by using the Resource Equation Approach.^[6]^ Briefly, when the tumor volume reached 100 mm^3^, the xenografted mice were randomly divided into four groups according to 5 mice per group, including the PBS group, PBS + L group, NPs group and NPs + L group. The mice in NPs group and NPs + L group were administered intravenously with SFA-BTM NPs (10 mg kg^-1^) for a single treatment. At 12 h post-injection, each mouse in the PBS + L group and NPs + L group was exposed to 808 nm laser (1W cm^-2^) once for 5 min. The 808 nm laser application was only localized to the primary tumor site. An *in vivo* imaging system was employed to monitor the changes in red fluorescence at tumor sites once every seven days, while the changes in body weight and tumor volume were tracked over a period of 21 days. On the 21st day, tumor paraffin sections were prepared from sacrificial mice for antitumor efficacy evaluation *via* haematoxylin and eosin (H&E) staining. The tumor volume measurements for the orthotopic model followed the previous method.^[7]^ The micro-CT images of tumor-bearing legs were obtained by a Siemens Biograph micro-CT device (Skyscan 1077, Antwerp, Belgium), the 3D models of mice tibias were reconstructed for observation in detail, and quantitative evaluation of bone repair was achieved by the CTVox programme (Bruker micro-CT NV, Antwerp, Belgium).

Pharmacokinetics study

The pharmacokinetics of SFA-BTM NPs were evaluated in SD rats (specific pathogen-free, SPF). Four SD rats were intravenous administration with of SFA-BTM NPs (10 mg kg^-1^) after precise weighing. At the present time points (0, 0.25, 0.5, 0.75, 1, 2, 4, 6, 8, 12 and 24 h), 250 μL blood of each rat was obtained and centrifugated at 15000 × g for 10 min to collect the plasma. After diluting 50-fold to 1 mL with 0.5 % acidified acetonitrile, the concentration of SFA-BTM in plasma samples was further quantified by HPLC/MS. The decay traces and fitting curve were provided. Area under the curve (AUC), mean retention time (MRT), terminal half-life (T_1/2_), and clearance (CL) were calculated.

*In vivo* biodistribution

The *in vivo* biodistribution study was conducted in 143B tumor-bearing BALB/c nude mice. Three tumor-bearing mice were intravenous administration with SFA-BTM NPs (10 mg kg^-1^). Then mice were sacrificed at 6, 12 and 24 h, and major organs and tumor tissue were collected. The *in vivo* biodistribution was decided by analysis concentration of SFA-BTM in each tissue *via* HPLC/MS.

**Biosafety Evaluation**

*In vivo* biosafety

After a 21-day treatment of the 143B tumor-bearing mice, paraffin sections of main organs were prepared from sacrificial mice and analysed by H&E staining. For further evaluation of biosafety, the treated mice were administered of blood routine and blood biochemistry analysis.

Biosafety evaluation of laser irradiation

To further assess the potential of skin burn damage, 6 healthy female mice without tumor burden were divided into two groups (n = 3 in each group): – laser group, + laser group. Then, all the mice were housed under standard conditions with free access to food and water. The mice in the + laser group received 808 nm laser irradiation under the same conditions as the tumor treatment study (1 W cm^-2^, 5 min) under anaesthesia. Following the requirement of Animal Ethical and Welfare, the mice were sacrificed at 24 h post-treatment. The laser-exposed and unexposed skin tissue was collected, and then H&E staining was performed. No skin burn damage was observed in the + laser group of mice. The animal experiments were approved by the Animal Care and Use Committee of Shenzhen Institute of Advanced Technology (SIAT).

Haemolysis test

500 μL fresh mouse blood was collected into an ethylenediaminetireaacetic acid (EDTA) functionalized tube and red blood cells (RBCs) were obtained by further centrifugation at 860 *× g* for 8 min. After discarding the supernatant, the remaining RBCs were washed with PBS until the supernatant became transparent. 100 μL resulted RBSc were diluted by 500 μL PBS and then 20 μL diluted RBCc was added into 480 μL water, PBS and PBS containing various concentrations of SFA-BTM NPs (6.25, 12.5, 25, 50, 100, 200 μg mL^-1^) as positive and negative controls as well as samples to be tested. The mixtures were vortexed and incubated at 37 ^o^C for 4 h. After 8 min of centrifugation at 860 × *g*, photograph the mixtures.

Enzyme activity evaluation

The Biocompatibility of SFA-BTM NPs on lipase, superoxide dismutase, acetylcholinesterase, and catalase were evaluated. The enzymes and related enzyme activity detection kits were purchased from Solarbio Life Science. After exposure to 25 μg mL^-1^ SFA-BTM NPs, enzyme activity was evaluated by protocol according to the manufacturer’s instructions.

1. **Supplementary Figures**

**Figure S1.** Synthetic route of DMA-BTM.

**Figure S2.** Synthetic route of DPA-BTM.

**Figure S3.** Synthetic route of SFA-BTM.


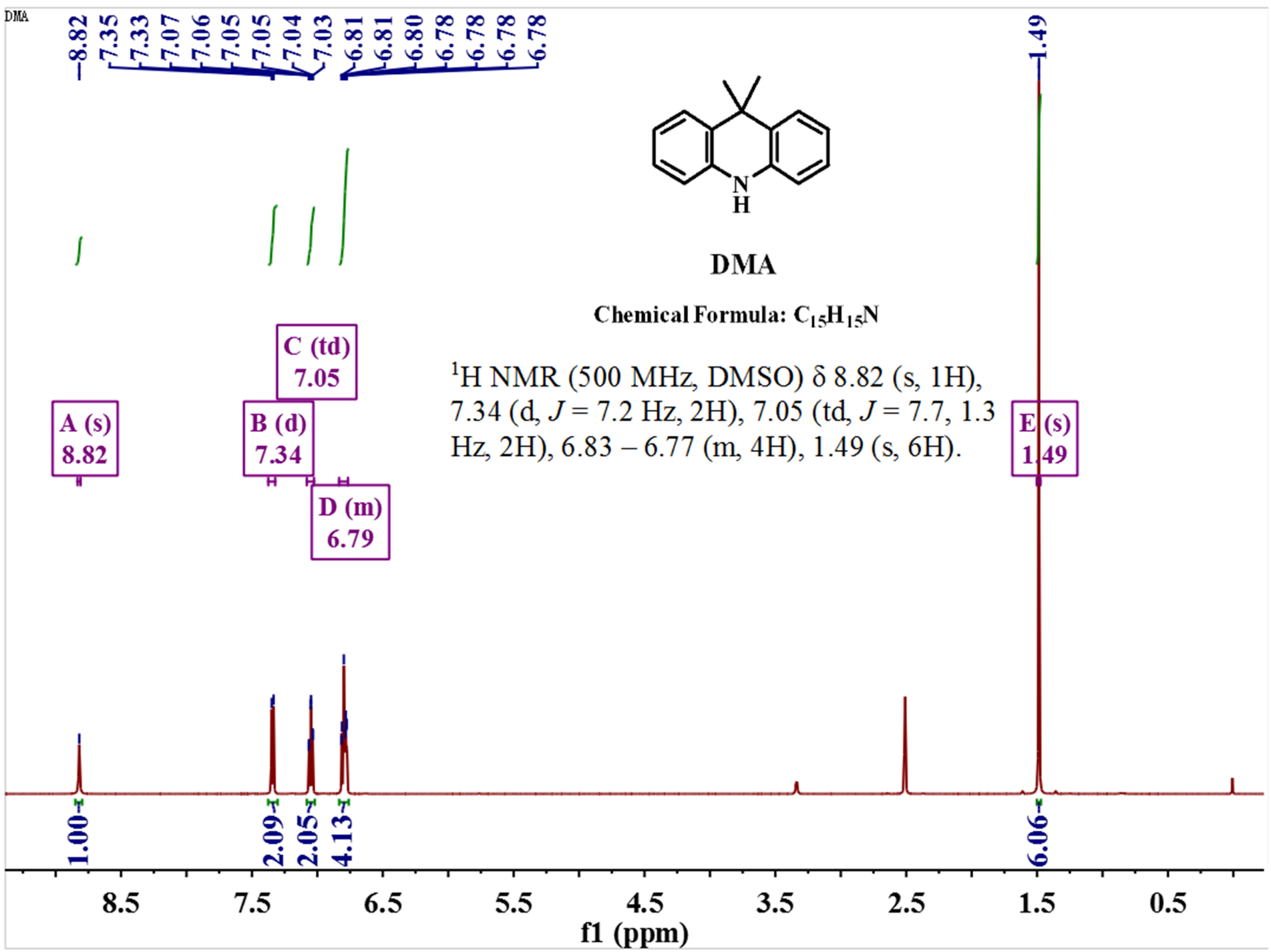


**Figure S4.** ^1^H NMR spectrum of DMA.


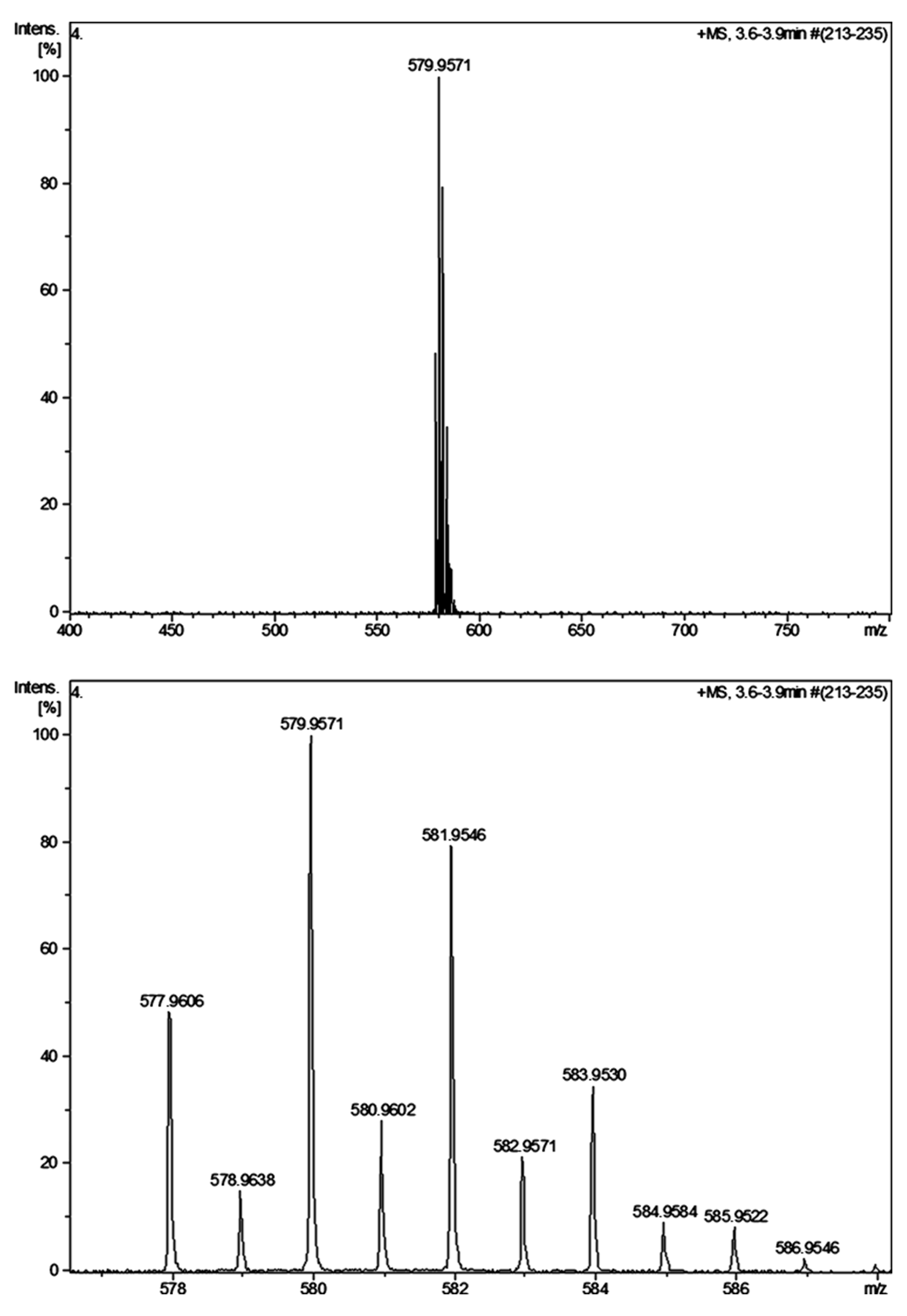


**Figure S5.** HRLC mass spectra of DMA-BTM.


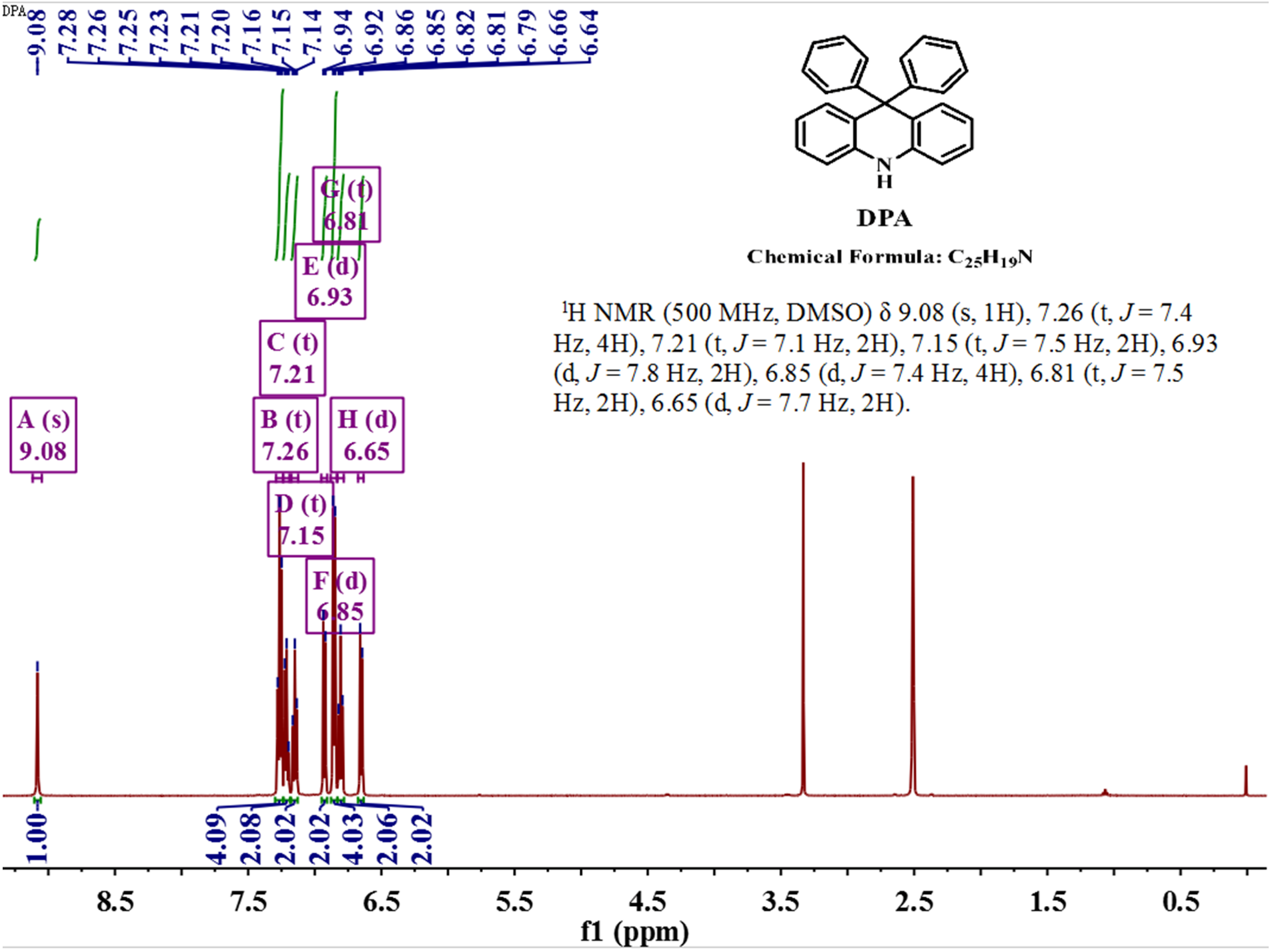

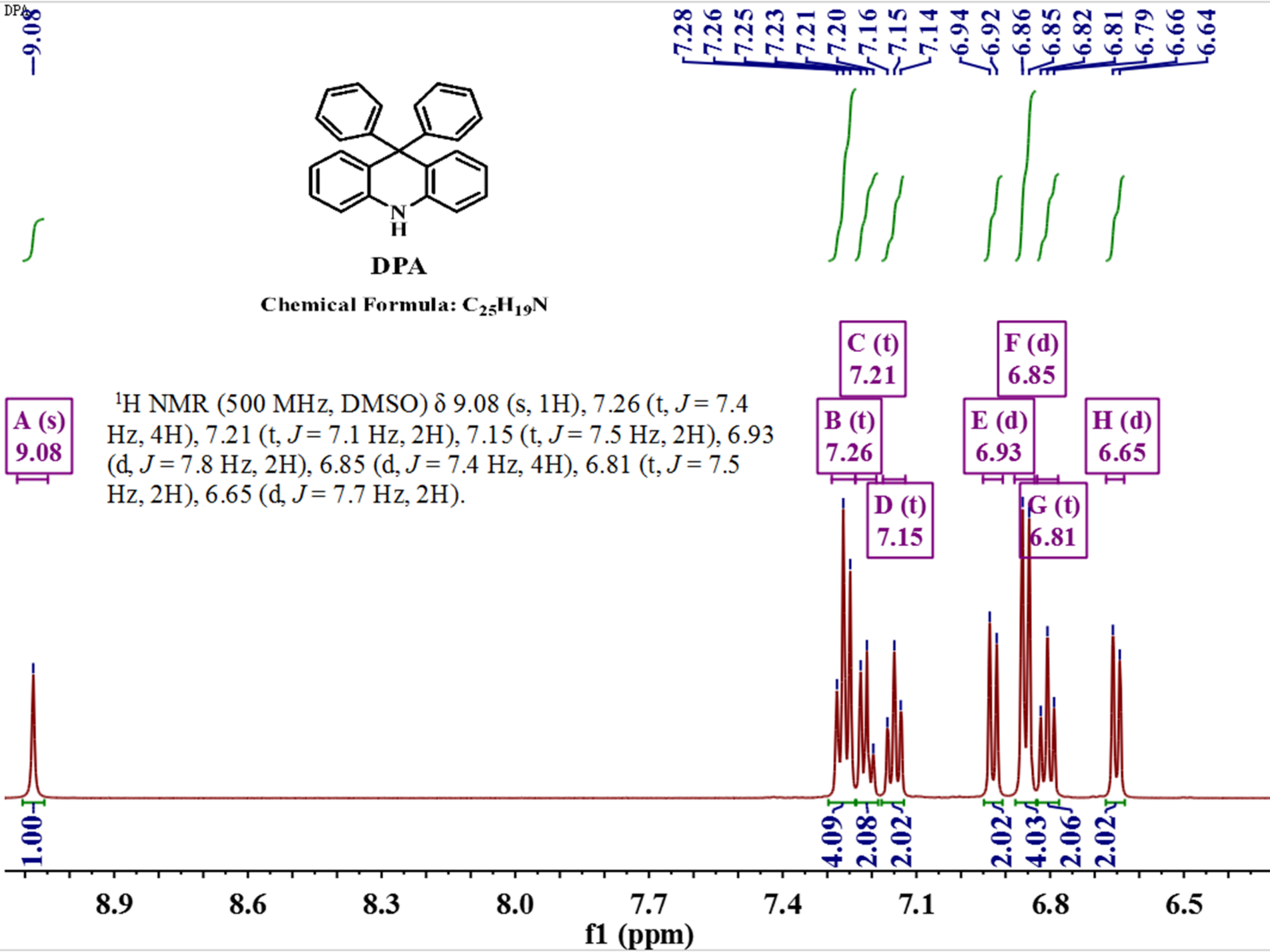


**Figure S6.** ^1^H NMR spectrum of DPA.


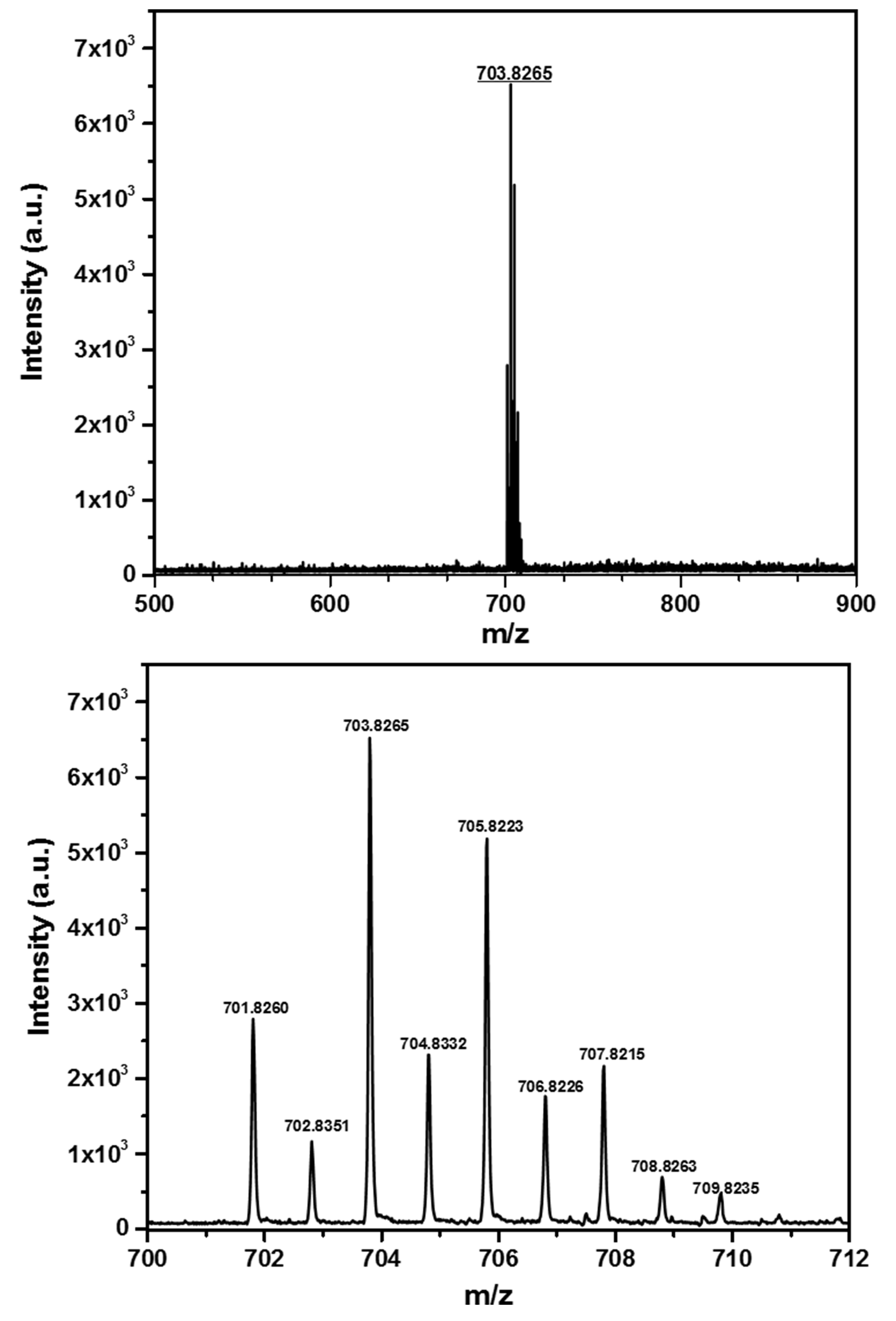


**Figure S7.** MALDI-TOF mass spectra of DPA-BTM.


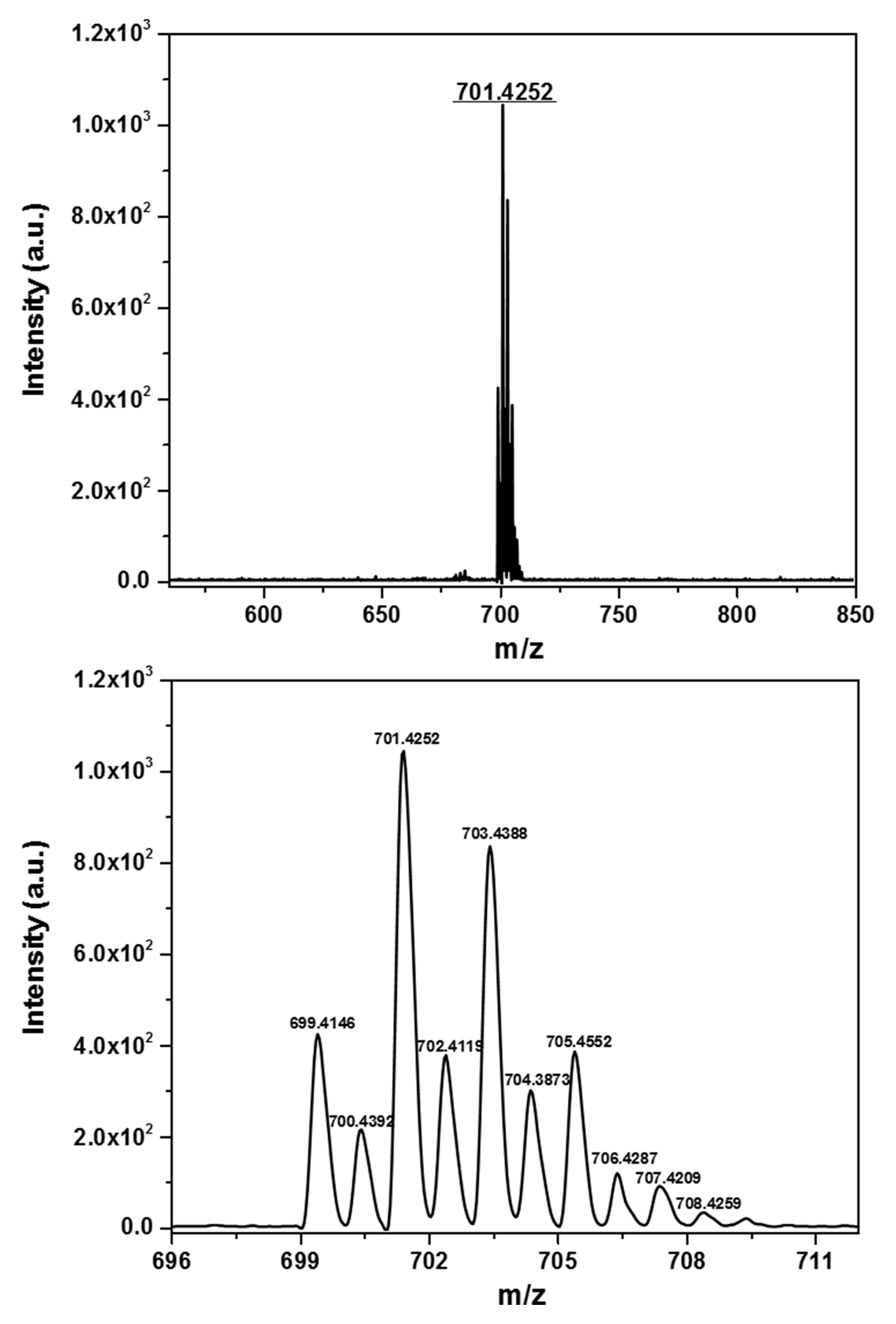


**Figure S8.** MALDI-TOF mass spectra of SFA-BTM.


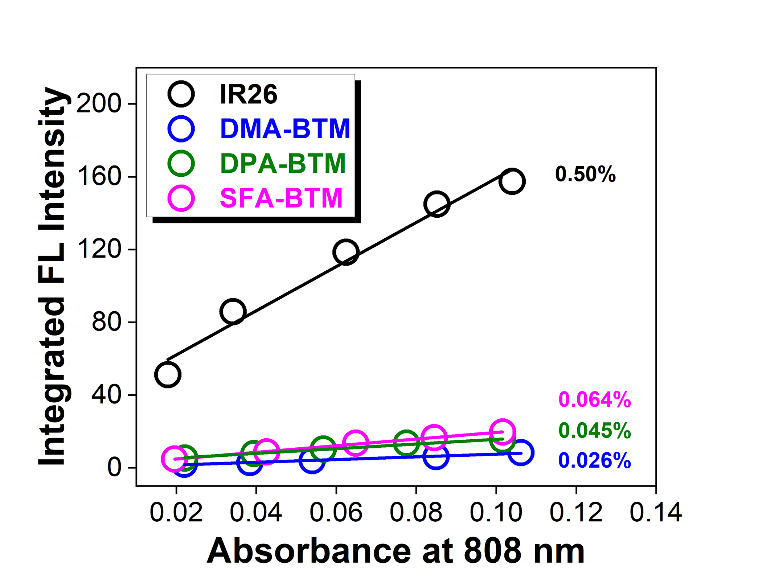


**Figure S9.** Relative fluorescence quantum yields (QYs) of SFA-BTM, DMA-BTM and DPA-BTM in tetrahydrofuran. IR26 was calculated as a QY of 0.5% in 1,2-dichloroethane as a reference. The excitation light source is 808 nm laser.


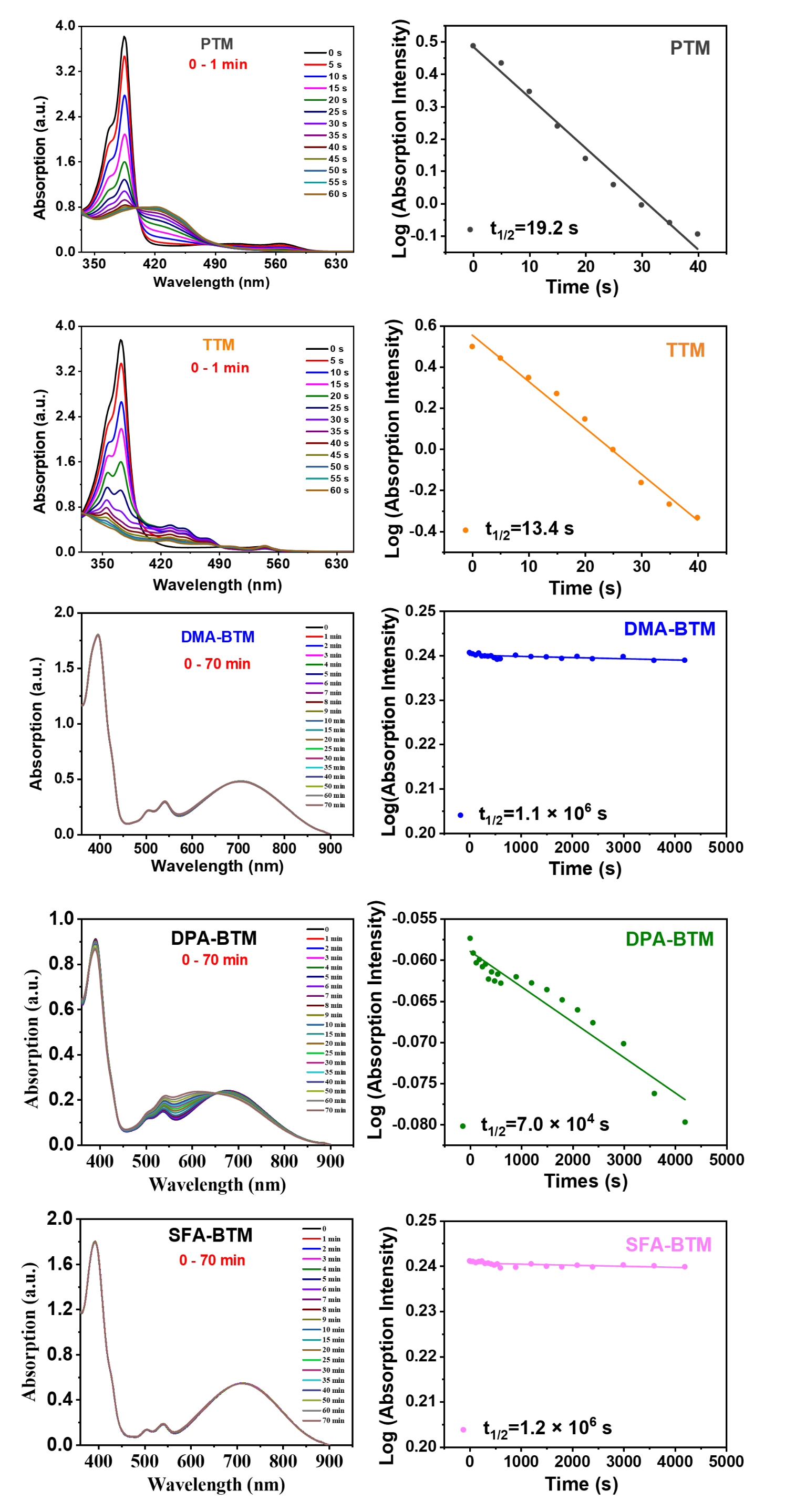


**Figure S10.** The photostability of neutral radicals is determined by characterizing their degradation velocity of their absorption spectra in dilute cyclohexane under 365 nm portable UV lamp irradiation.


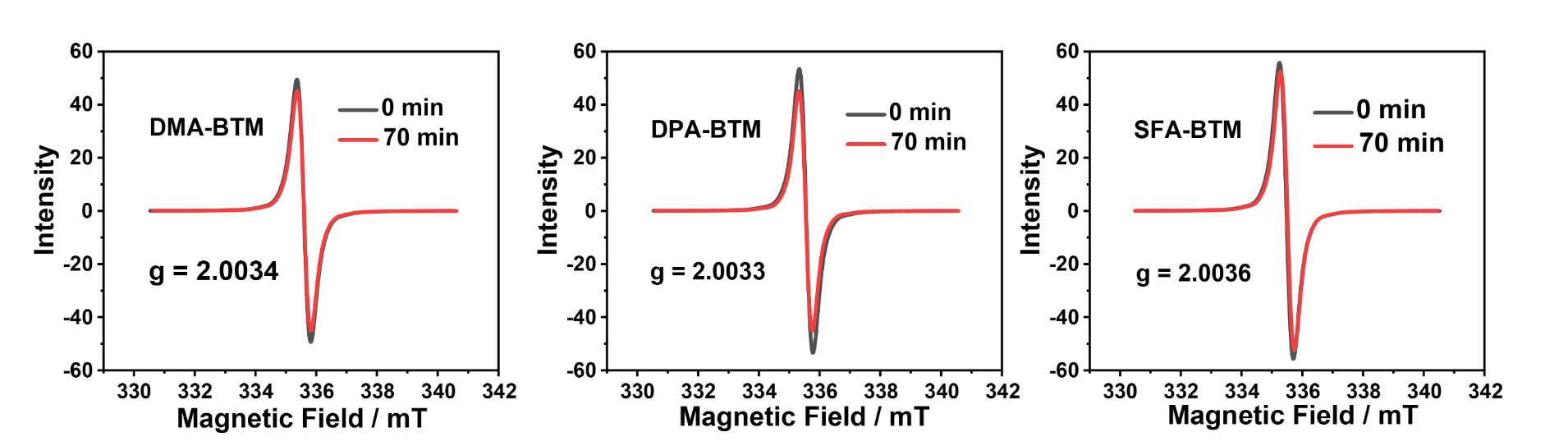


**Figure S11.** EPR spectra of three radicals (100 μmol L^-1^) in toluene before and after 365 nm irradiation. The power density of the UV lamp was 1.3 W cm^-2^.


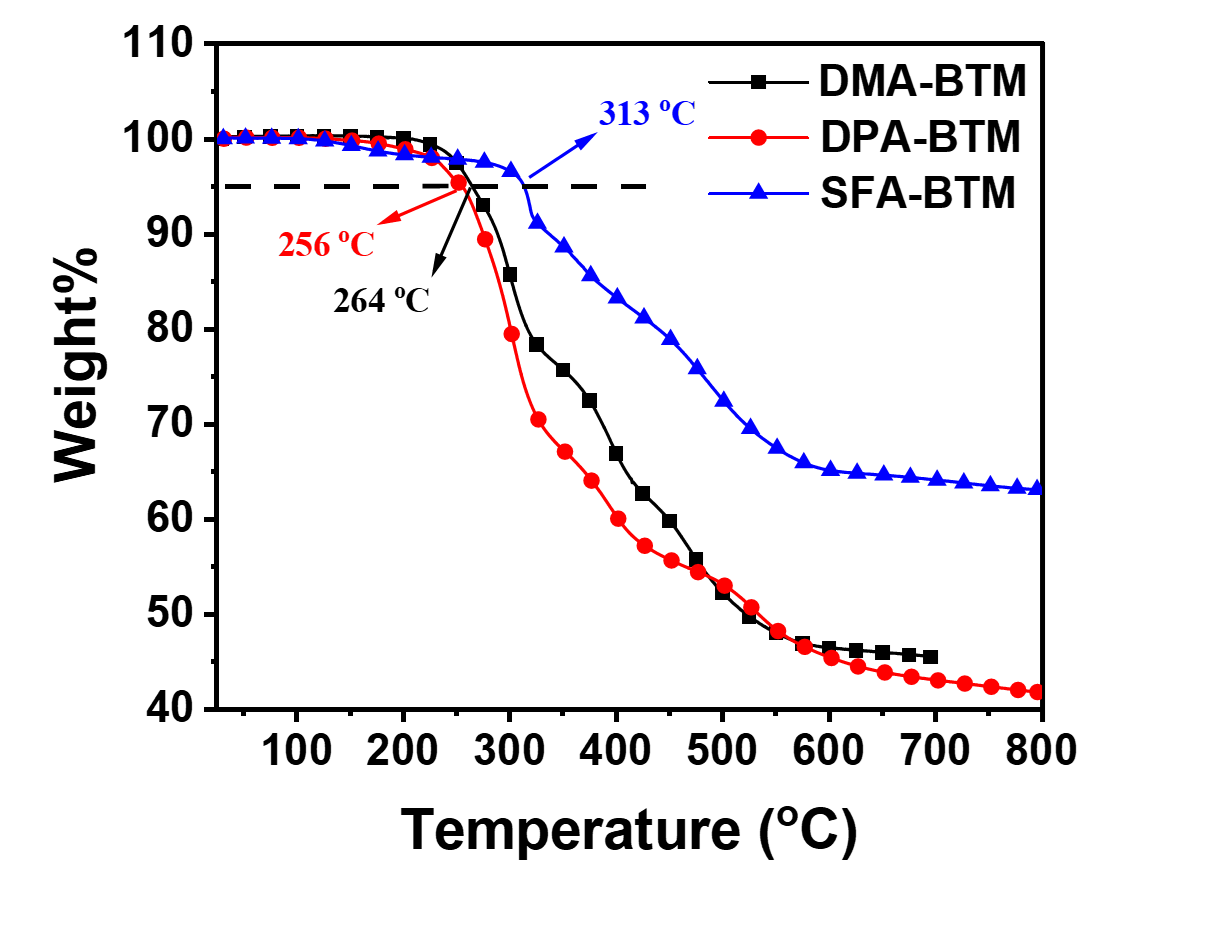


**Figure S12.** TGA curves of DMA-BTM, DPA-BTM and SFA-BTM under nitrogen flow.


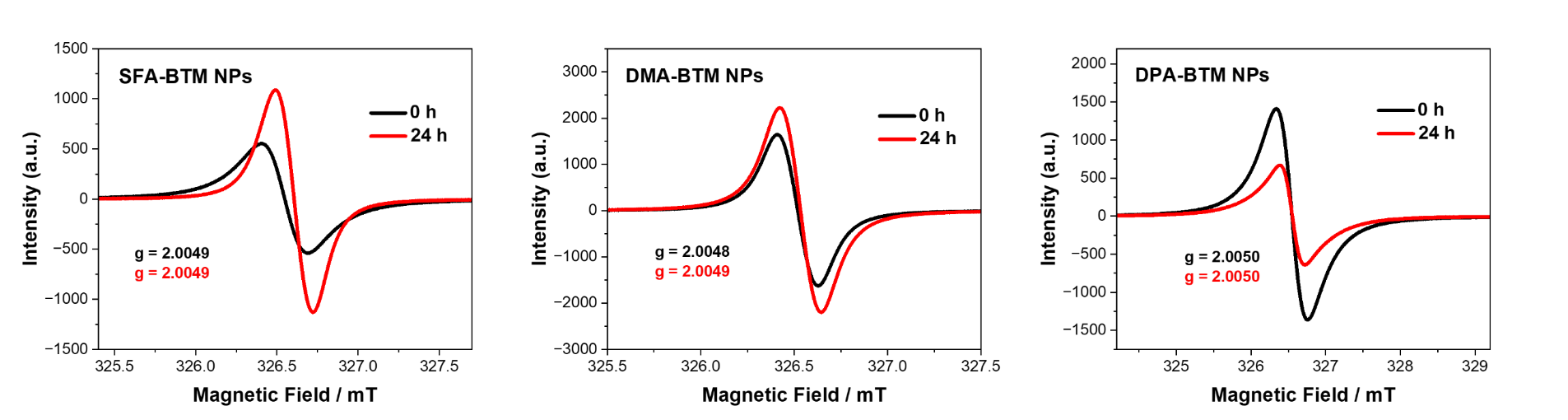


**Figure S13.** EPR signals of D-A radical nanoparticles before and after 24 h incubation in boiling water.


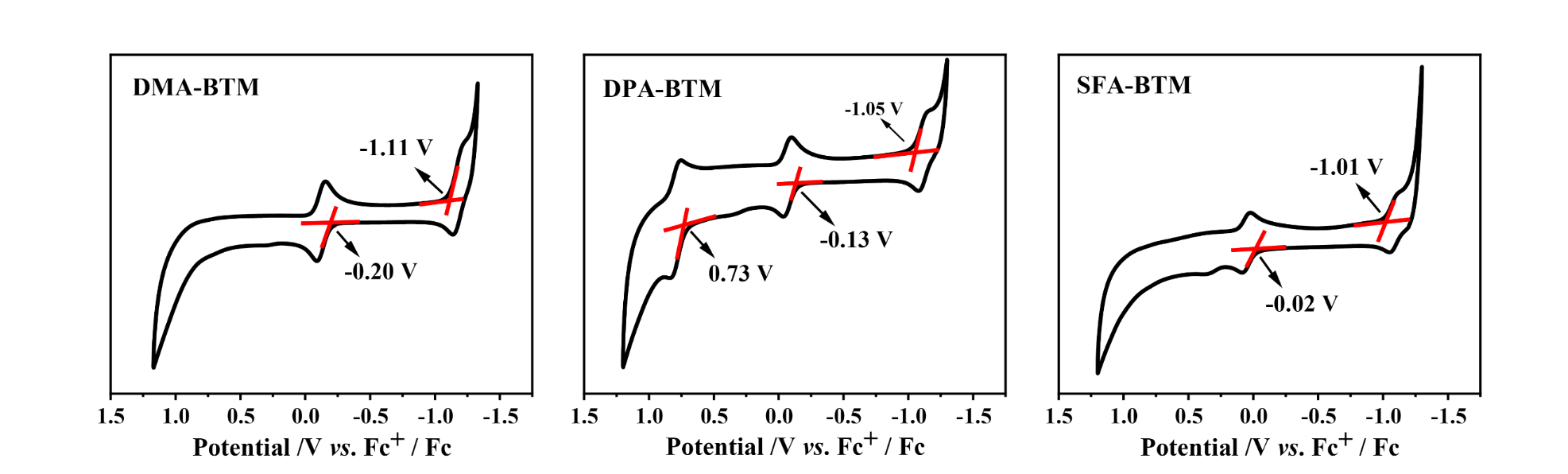


**Figure S14.** Cyclic voltammograms of DMA-BTM, DPA-BTM and SFA-BTM in CH_2_Cl_2_ at room temperature using 0.1 M *n*Bu_4_PF_6_ as a supporting electrolyte. Ag/Ag^+^ was used as the reference electrode and the ferrocenium/ferrocene redox couple was used as an internal standard. The corresponding orbital levels of three radicals were calculated using the equation E_orbital_=-e(E_ox/red_+4.8) (eV) and listed in Table S11-12.^[8]^

**
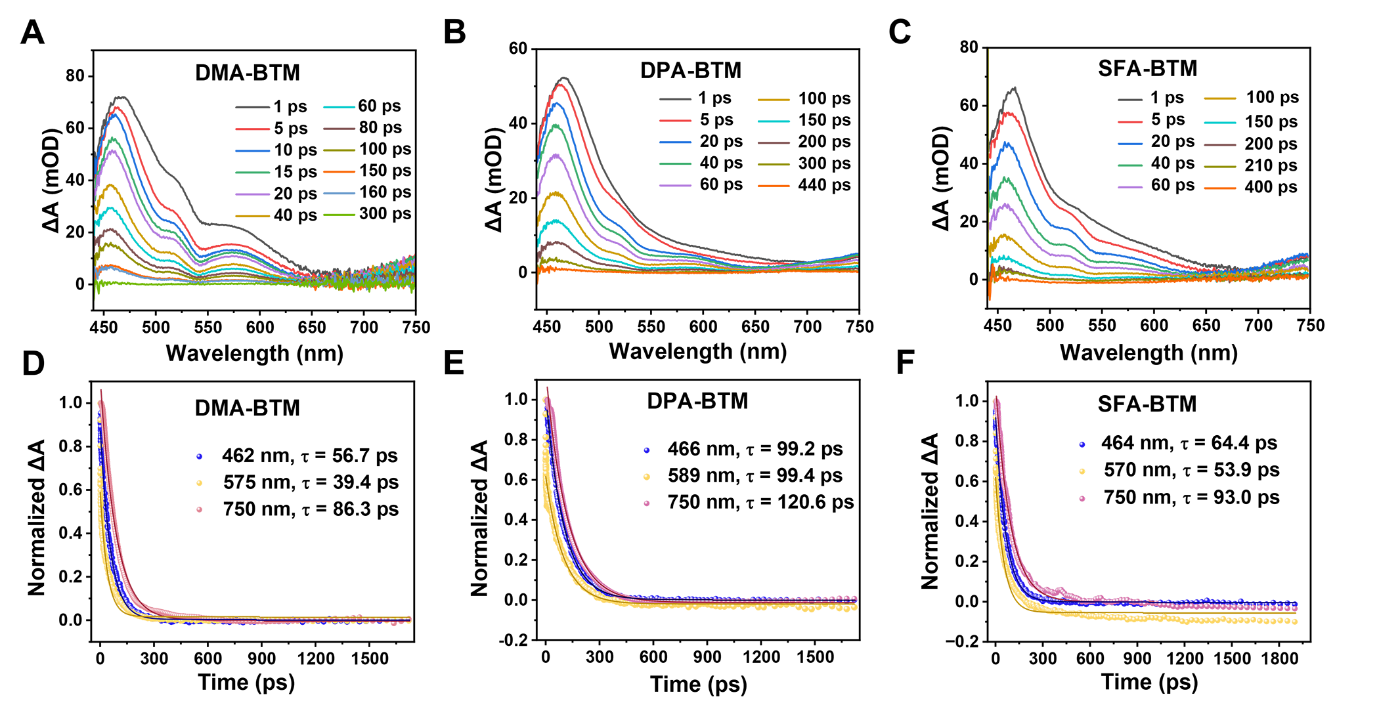
**

**Figure S15.** Transient absorption spectra of (**A**) DMA-BTM, (**B**) DPA-BTM and (**C**) SFA-BTM in toluene with concentration of 100 μmol L^-1^. (**D**-**F**) The corresponding decay curves of the three radicals. The excitation laser wavelength was 390 nm.


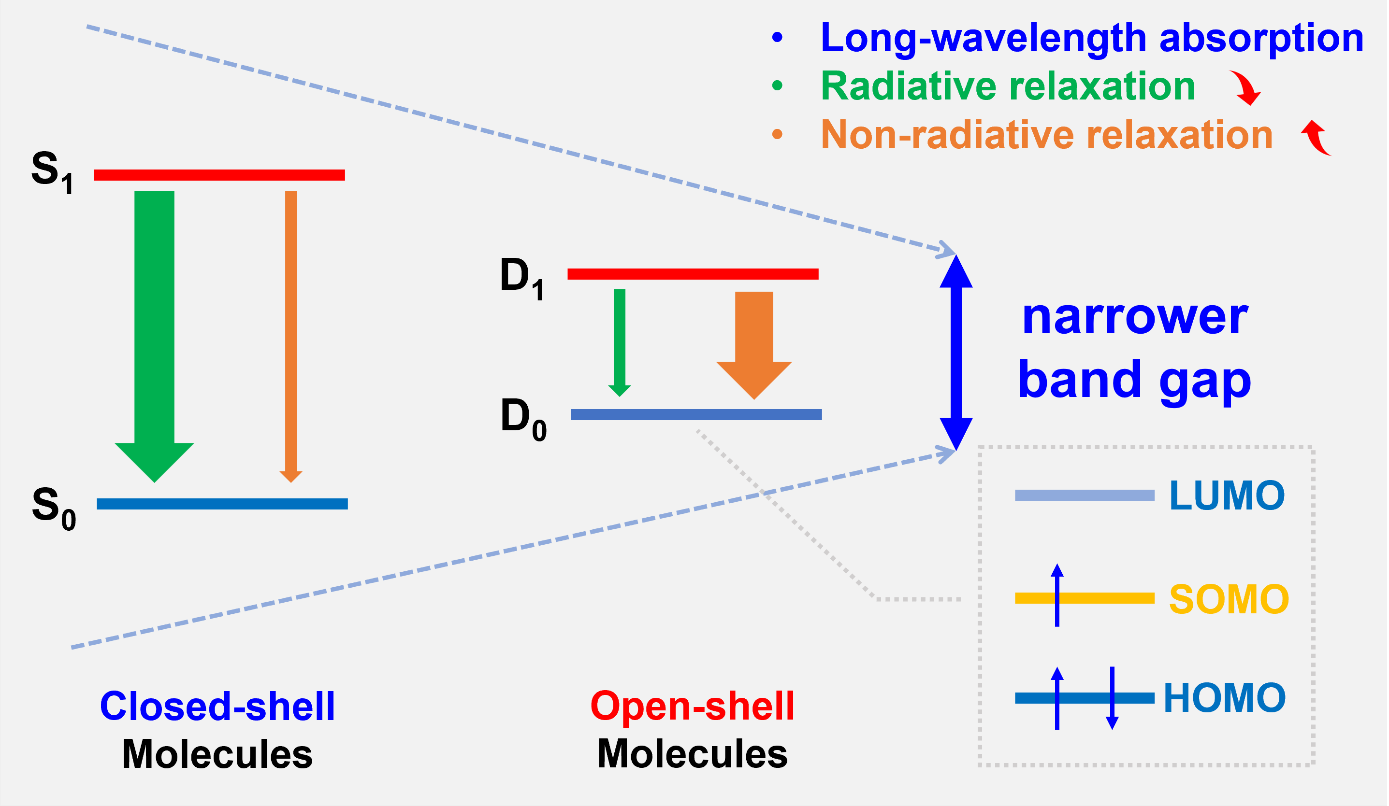


**Figure S16.** Schematic illustration of advantages of the organic radicals with open-shell performance as photothermal agents.

**
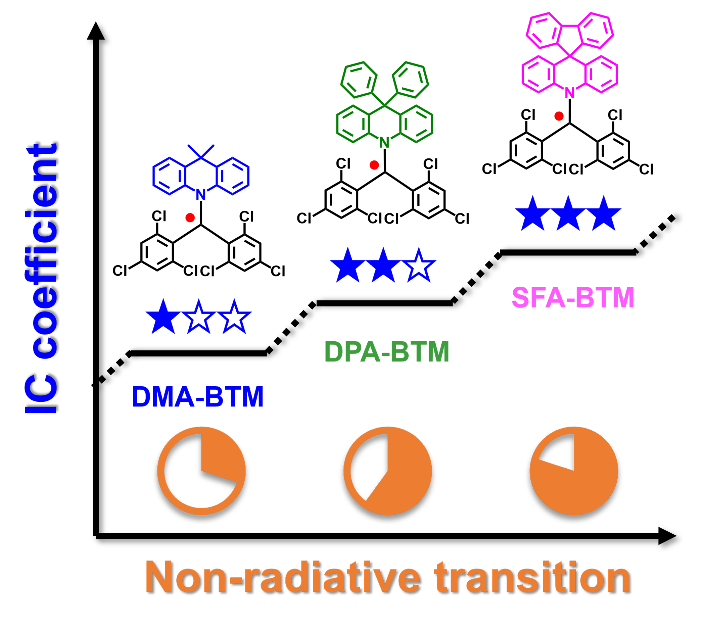
**

**Figure S17.** Schematic illustration of the relationship between the internal conversion (IC) coefficient and the non-radiative transition of these D-A radicals.


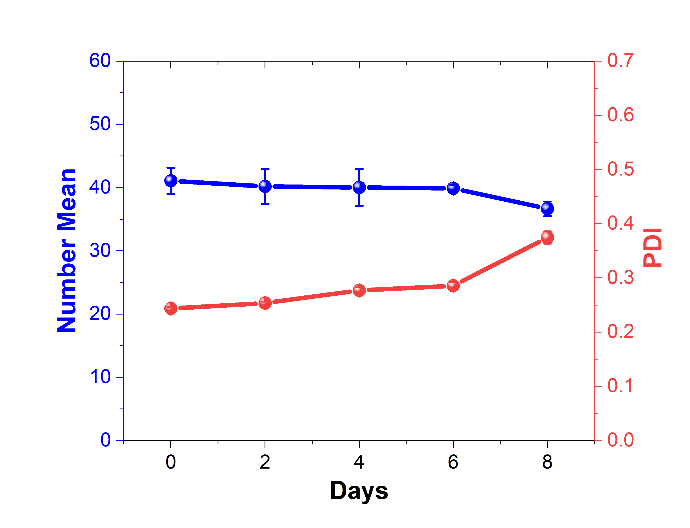


**Figure S18.** Dispersion stability of SFA-BTM NPs over 8 days. Data was presented as mean ± standard deviation, calculated based on data from three replicates of the experiment.


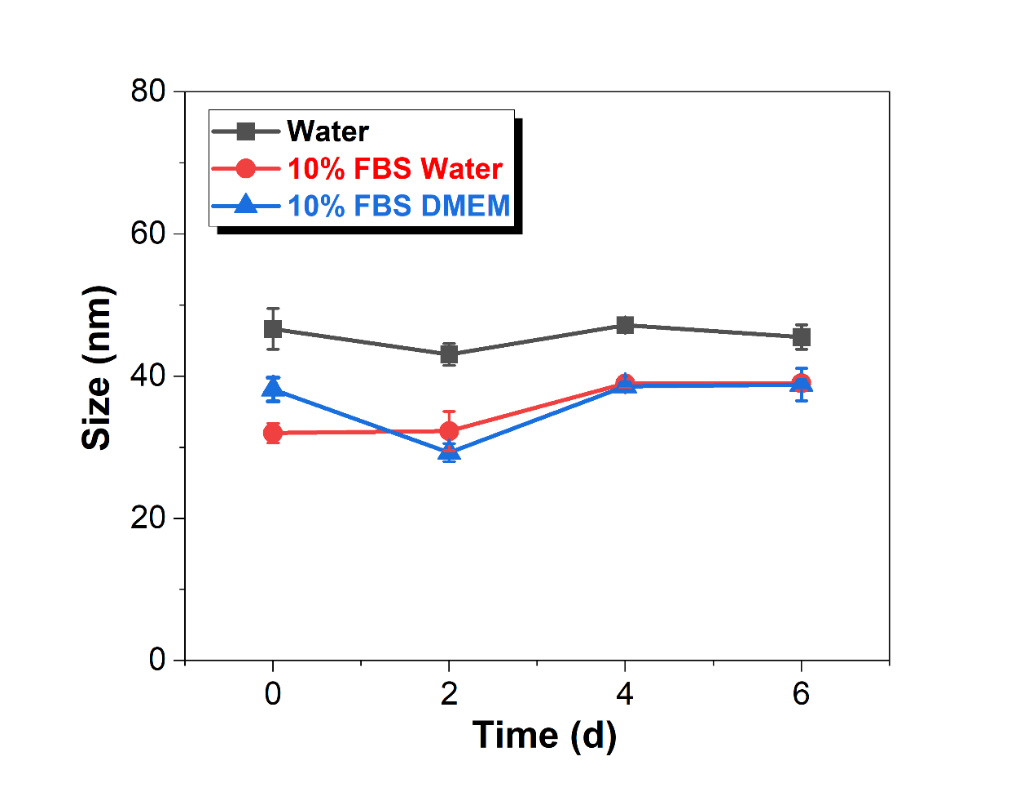


**Figure S19.** Size stability of SFA-BTM NPs in different physiological environments over 6 days. Data were presented as mean ± standard deviation, which was calculated from the data of three replicates of the experiment.

**
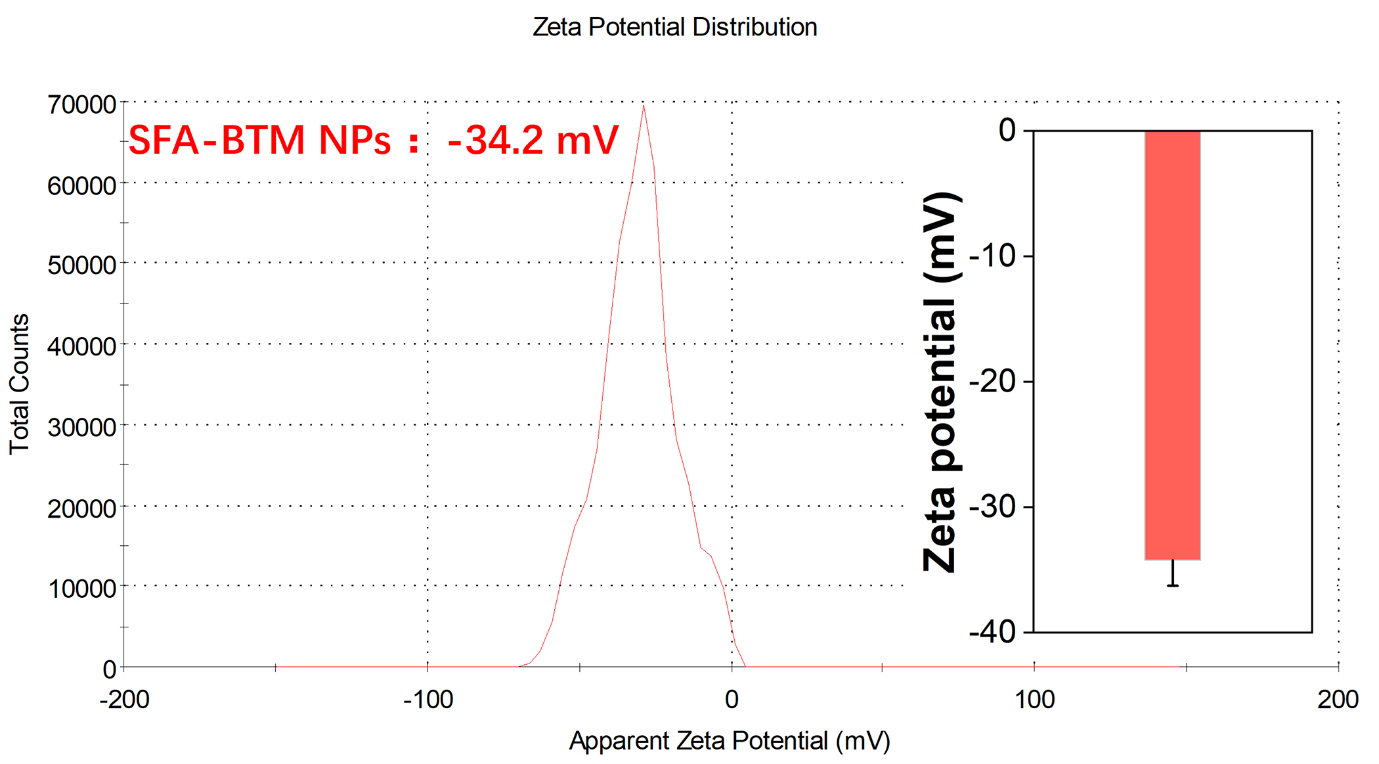
**

**Figure S20.** Zeta potential of SFA-BTM NPs in aqueous solution. Data were presented as mean ± standard deviation, n = 3.

**Figure S21.** The emission spectra of molecular SFA-BTM in THF and SFA-BTM NPs in aqueous solution with the same OD of 0.1 at 808 nm. The excitation light source is 808 nm laser.

**
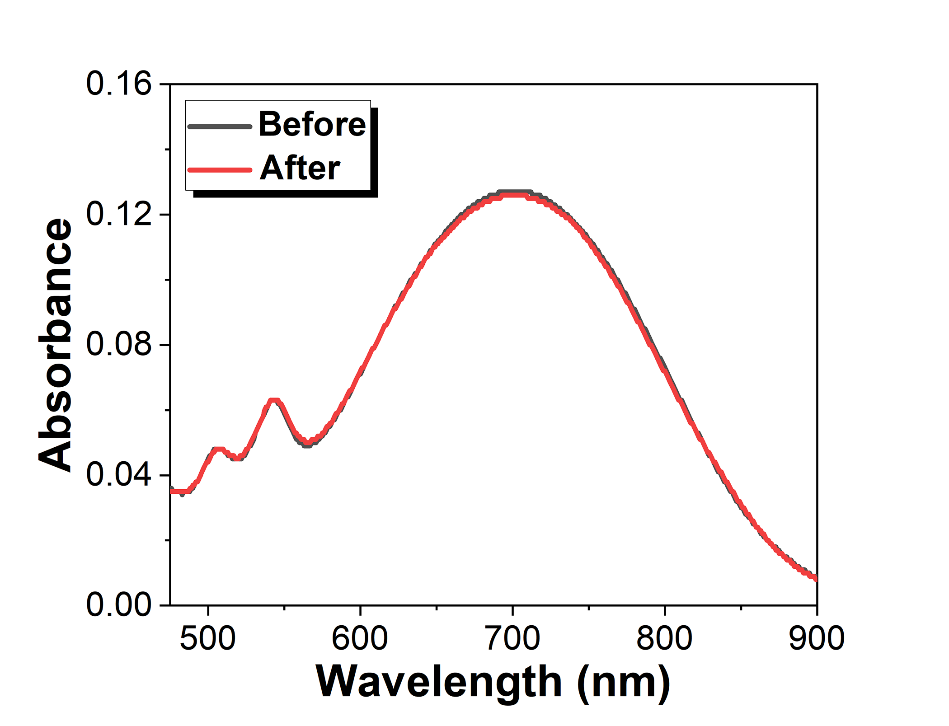
**

**Figure S22.** The absorption spectra of SFA-BTM NPs (50 μg mL^-1^) before and after 10 min of 808 nm laser irradiation in water. Power density: 1 W cm^-2^.

**
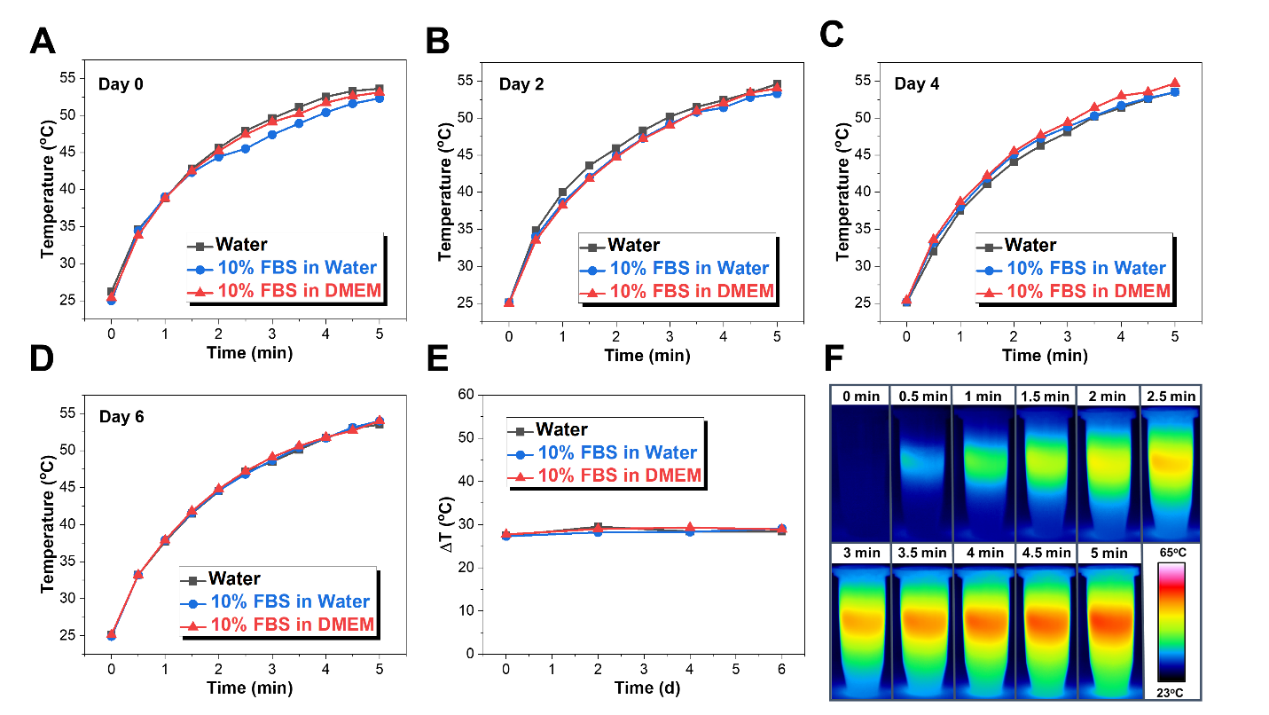
**

**Figure S23.** Photothermal stabilities of SFA-BTM NPs in different physiological environments as a function of incubation time. (**A-D**) Temperature rise of SFA-BTM NPs (100 μg mL^-1^) in water, 10% FBS in water, and 10% FBS + DMEM at different times. (**E**) Photothermal stability of SFA-BTM NPs in different environments over 6 days. (**F**) Infrared thermal images of SFA-BTM NPs in 10% FBS + DMEM. The evaluation was conducted under an 808 nm laser (1 W cm^-2^) at room temperature.


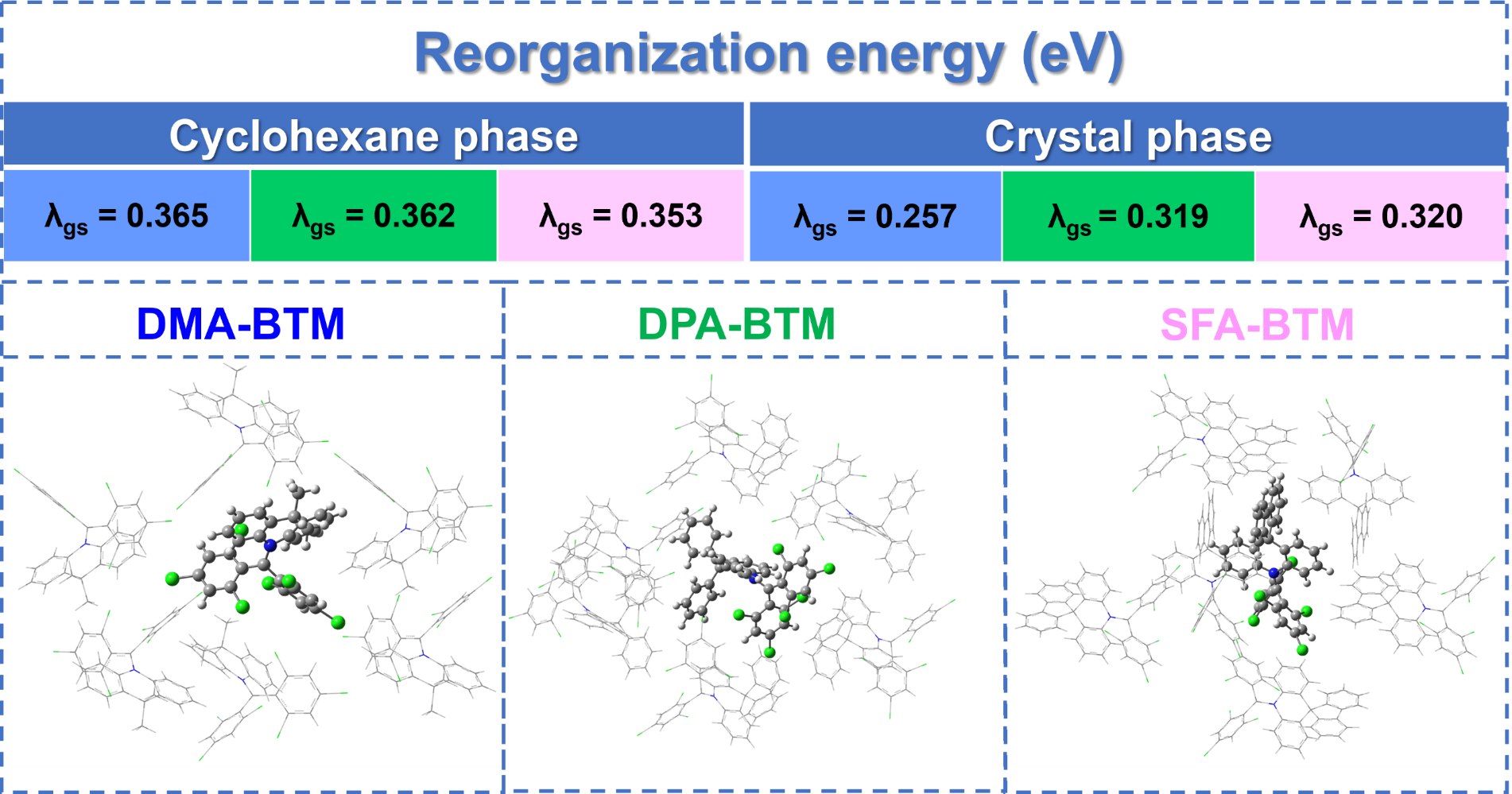


**Figure S24.** The reorganization energies calculated in the cyclohexane and crystal phases. The data were calculated using ONIOM(B3LYP/6-31+G(d,p) :UFF)-EE model in the crystal phase.


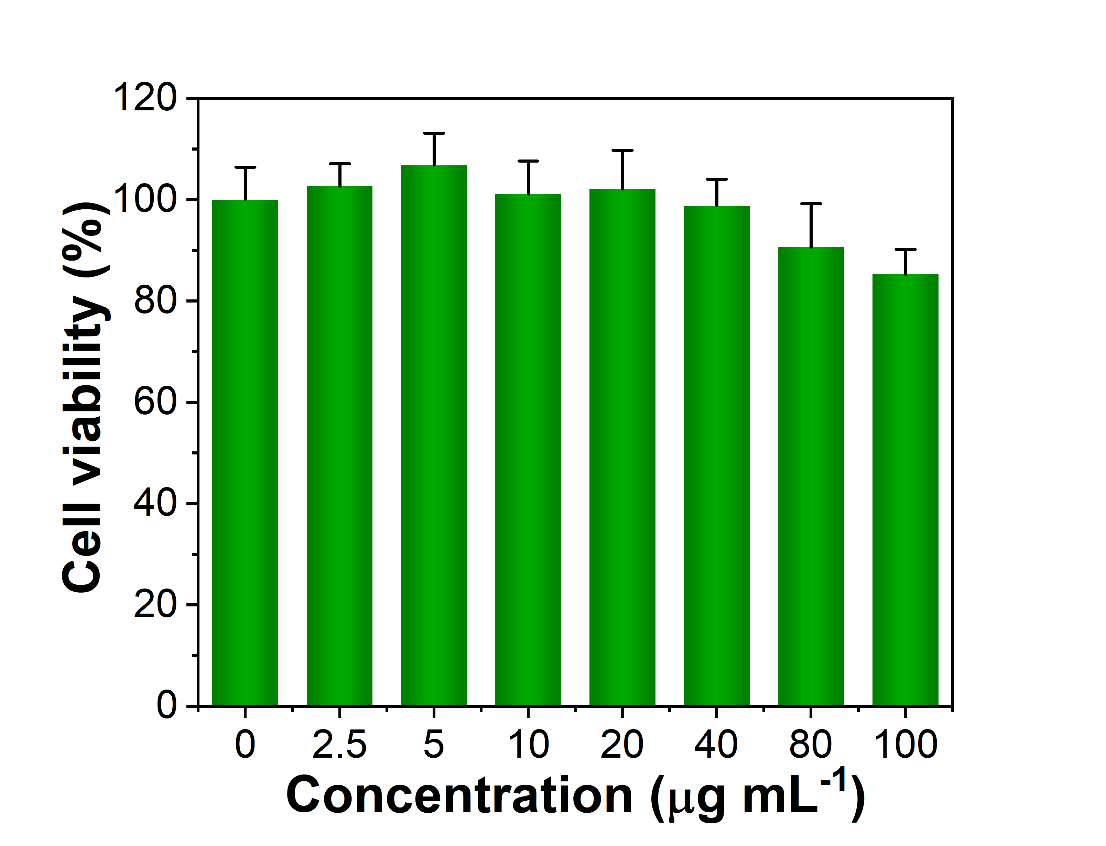


**Figure S25.** Cell viability of NIH-3T3 cells after treated with SFA-BTM NPs at different concentrations. Data was presented as mean ± standard deviation, which was calculated from the data of three biologically independent samples.


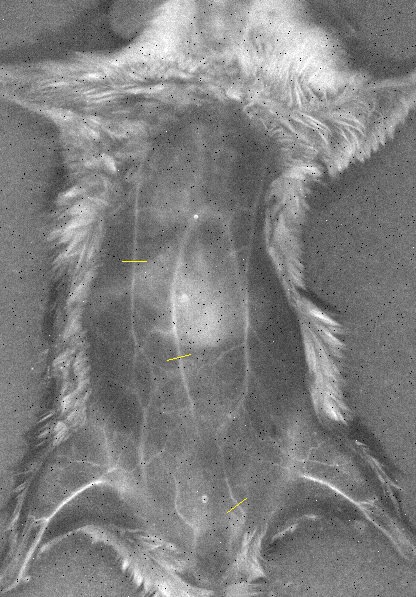


**Figure S26.** Whole-body NIR bioimaging of the mouse with 1500 nm long-pass (LP) filters.


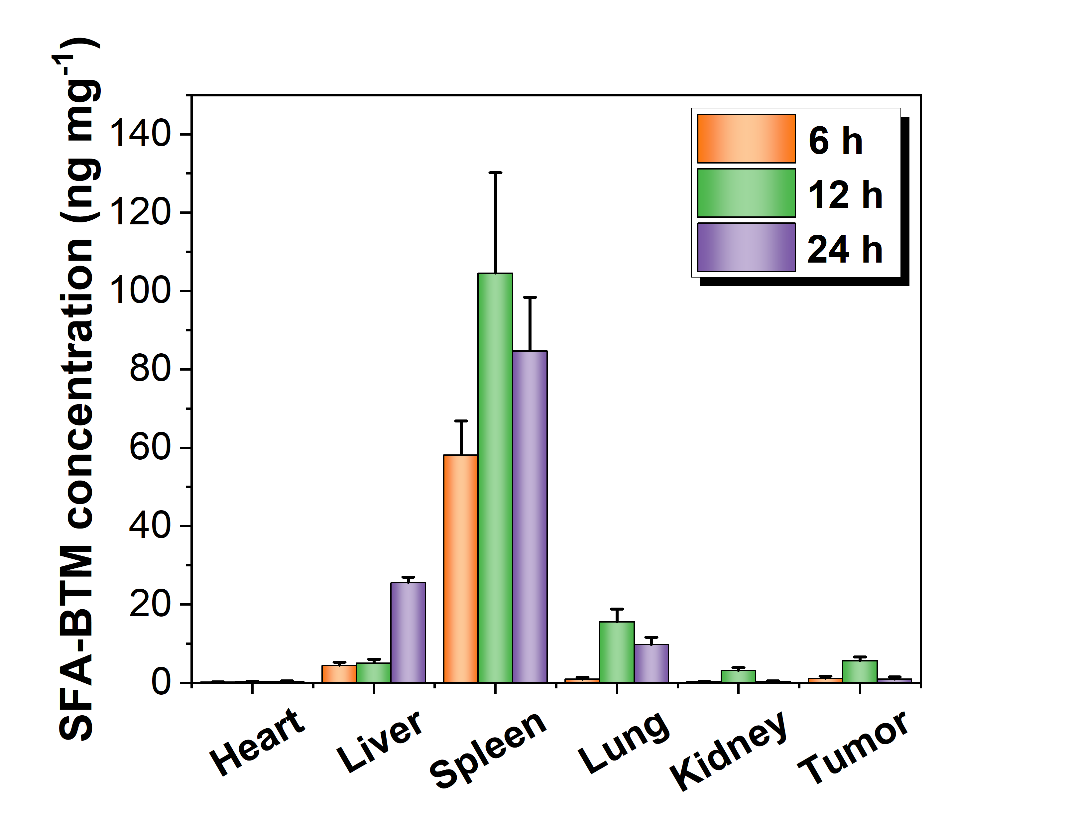


**Figure S27.** *In vivo* biodistribution of SFA-BTM NPs in major organs and tumor tissue in different post-injection times. Data were presented as mean ± standard deviation, calculated based on data from three biologically independent mice.


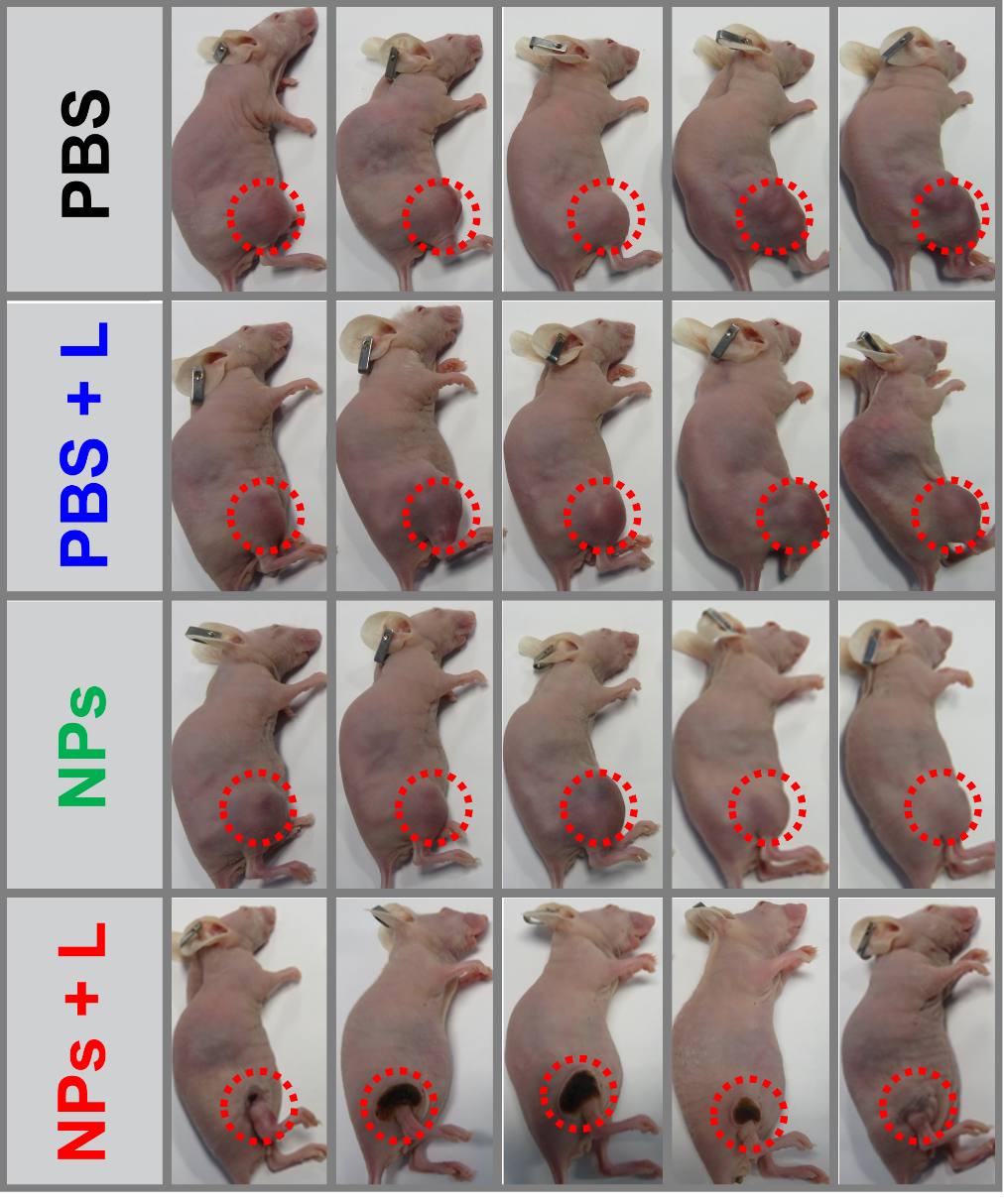


**Figure S28.** The tumor photographs representing each biological independent mouse in various treatment groups on day 21 post PDT.


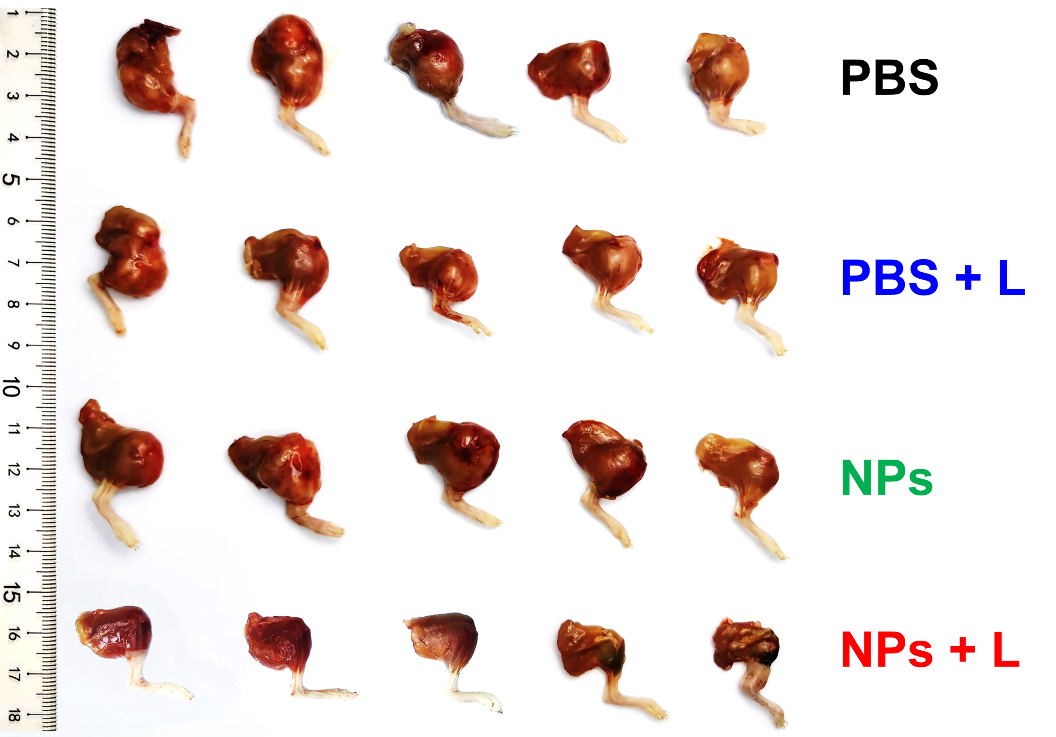


**Figure S29.** Photographs of the excised tumors after 21 days of growth in different treatment groups. From left to right were the tibia of five mice in each treatment group.


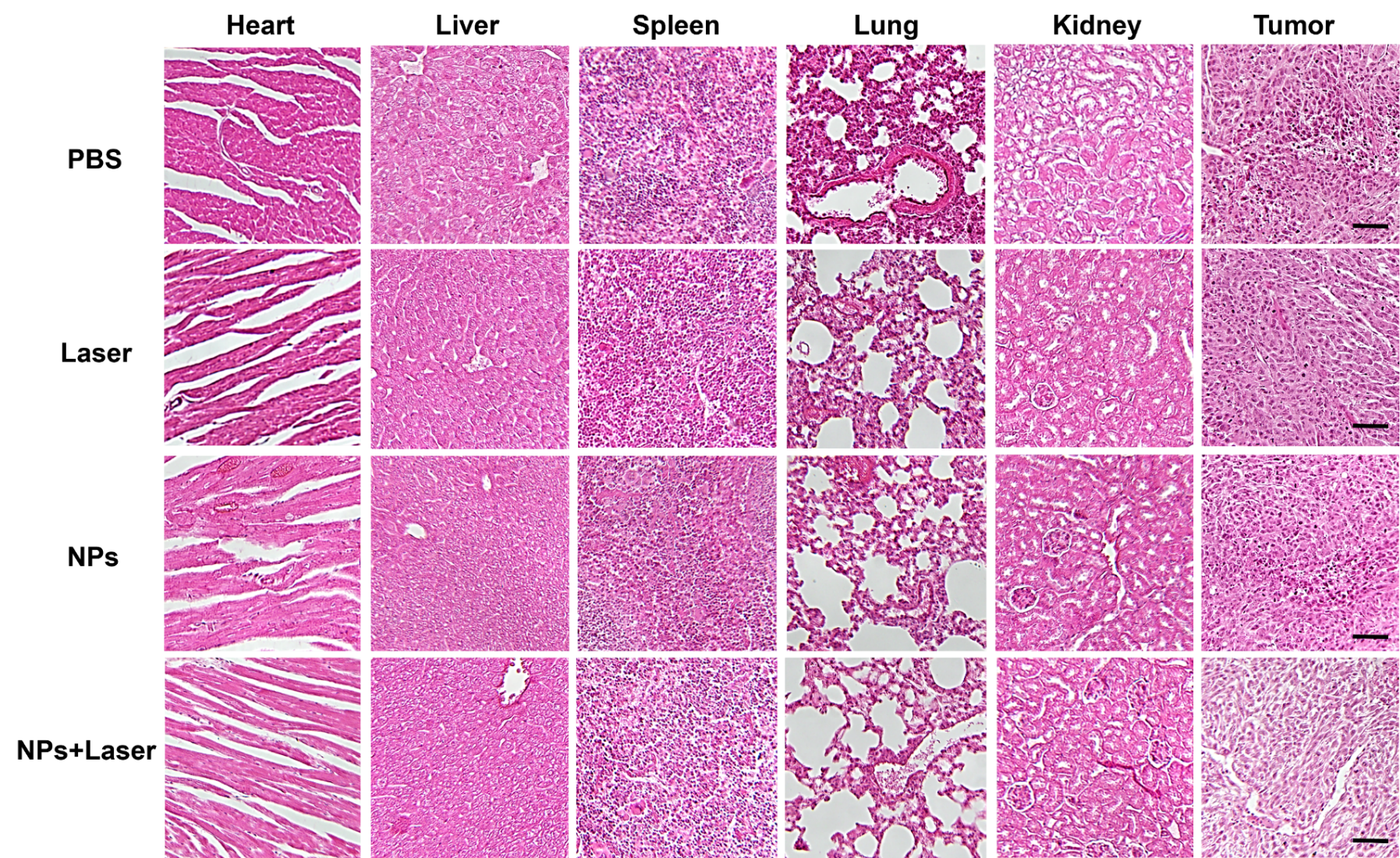


**Figure S30.** H&E staining images of tumor tissue and major organs from sacrificial mice. The scale bar is 100 μm.


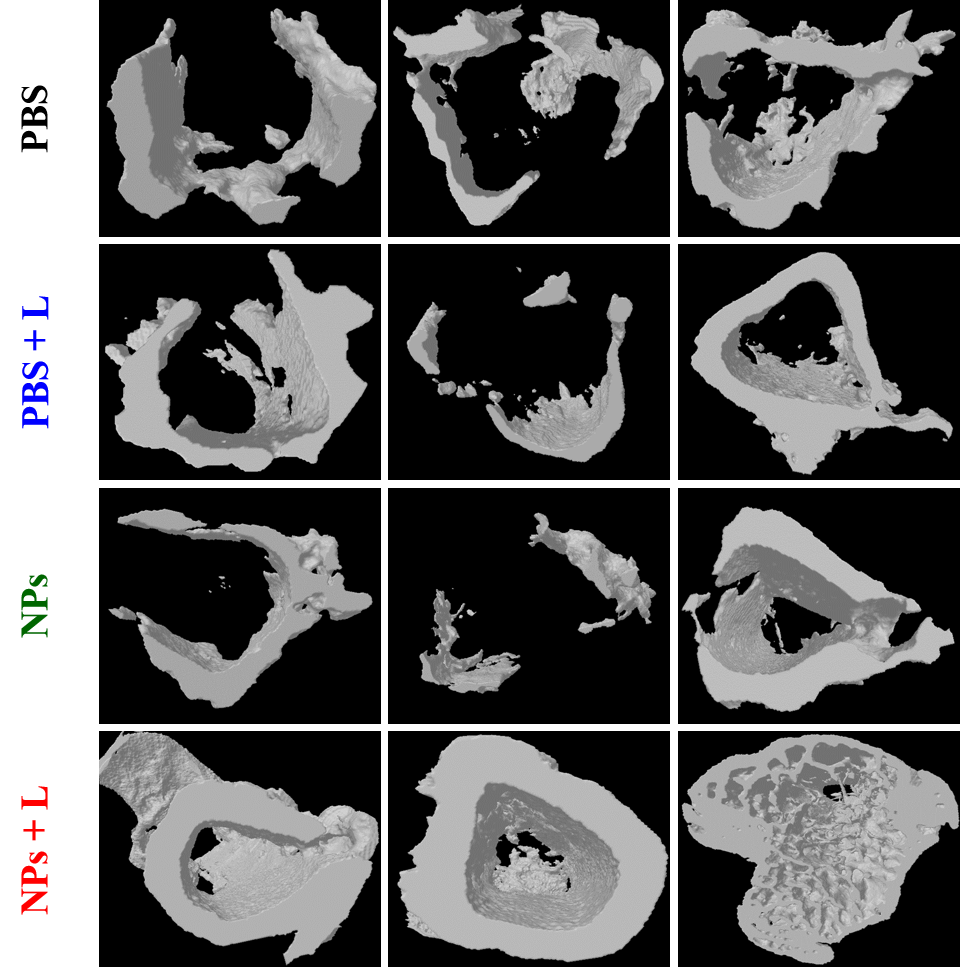


**Figure S31.** The 3D profiles of tibia fragments from different groups. The CT images from left to right represented the tibia fragments from each mouse in the same treatment group.


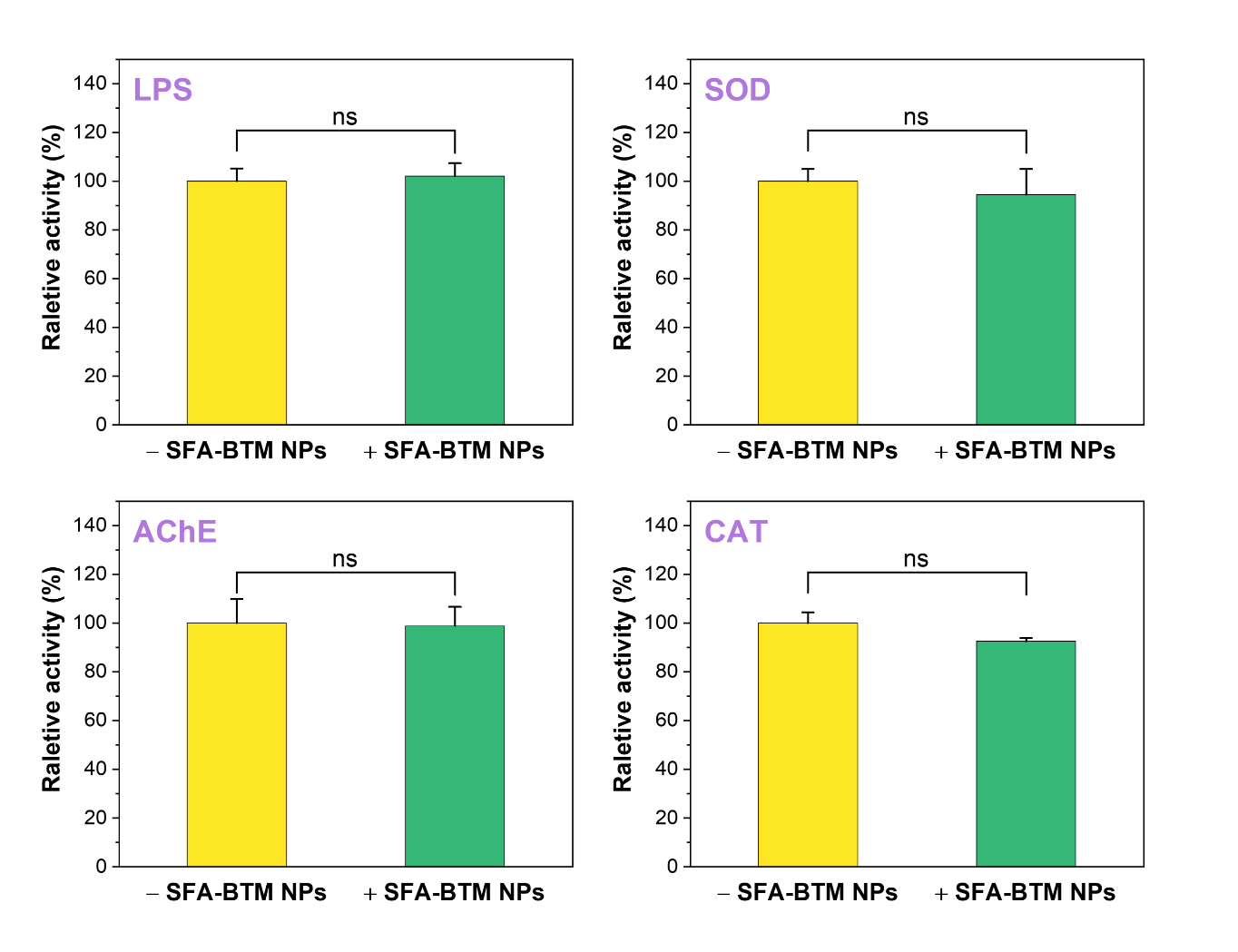


**Figure S32.** Biocompatible evaluation of SFA-BTM NPs (25 μg mL^-1^) towards enzymes. LPS: Lipase, SOD: Superoxide dismutase, AChE: Acetylcholinesterase, CAT: Catalase. Data from three replicates were presented as mean ± standard deviation, n = 3. The statistical difference was calculated using one-way ANOVA with Tukey’s test. ns: non-significant differences.

**
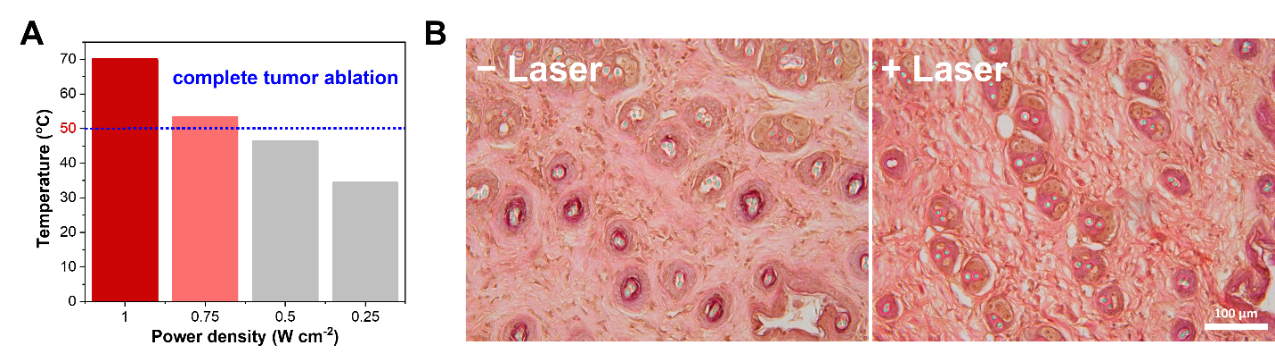
**

**Figure S33.** The justification of power density. (**A**) Representative the temperature comparison of SFA-BTM NPs (200 μg mL^-1^) exposed to 808 nm laser at various power densities for 10 min. (**B**) Evaluation of biosafety of 1 W cm^-2^ power density to the skin for 5 min irradiation using H&E staining. Scale bar: 100 μm.


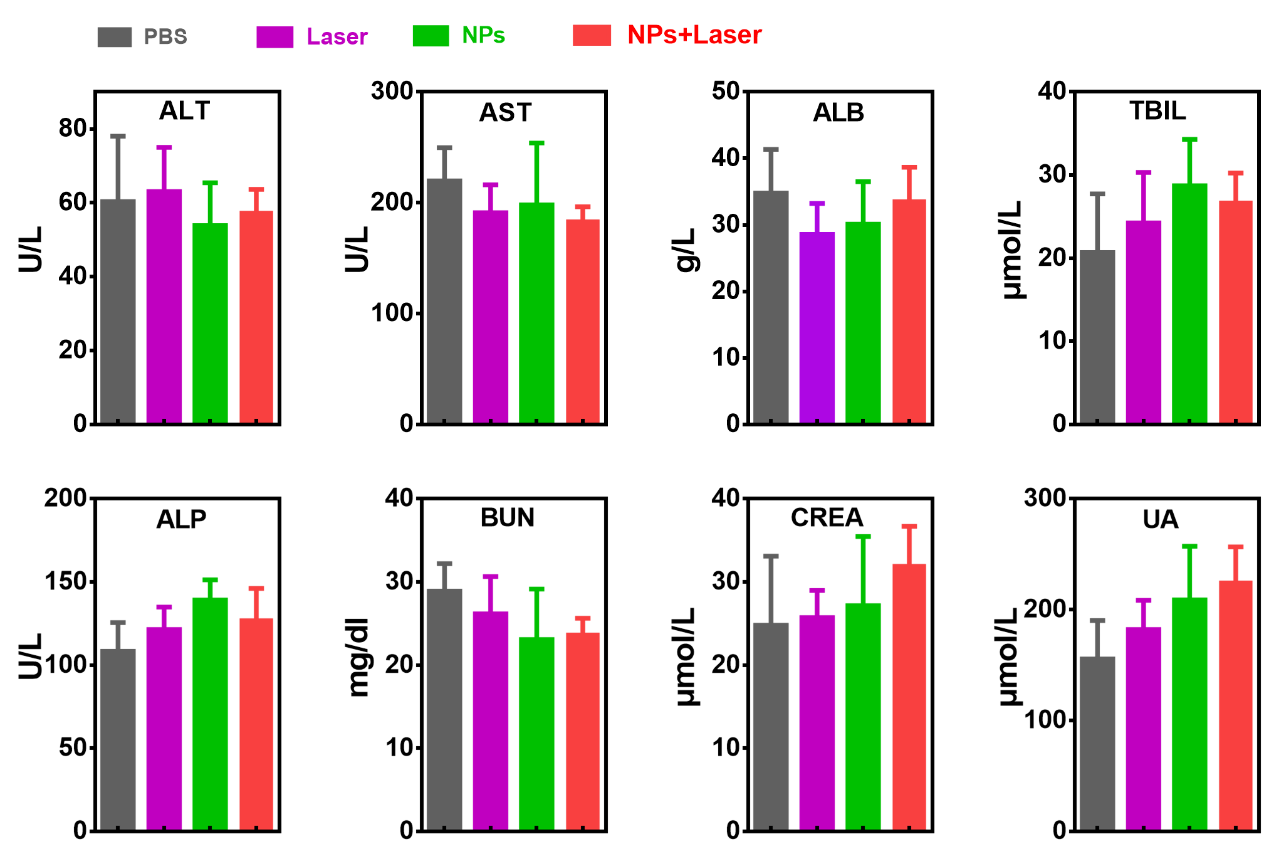


**Figure S34.** Hepatic and renal function evaluation by blood biochemistry analysis of mice after various treatments. Data was presented as mean ± standard deviation, which was calculated from the data of three biologically independent mice.


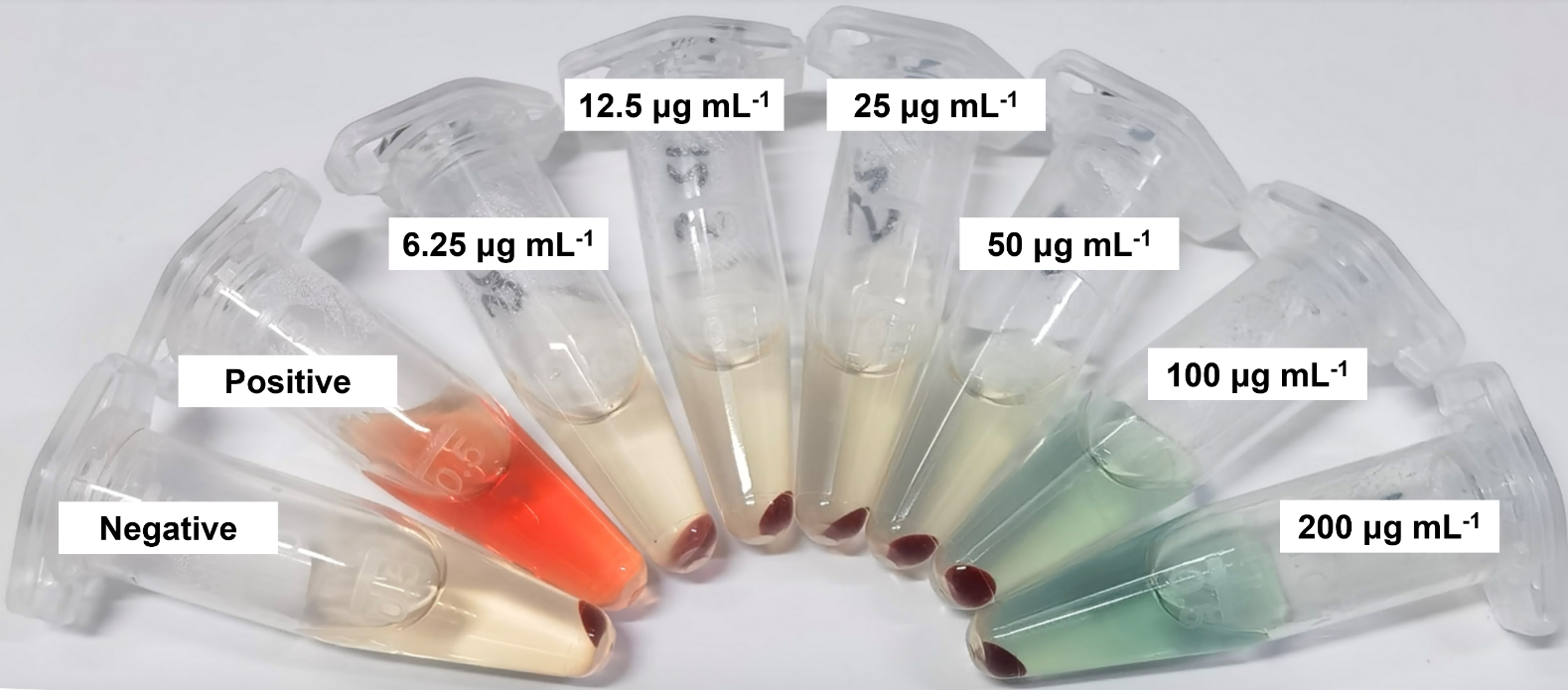


**Figure S35.** Hemolytic images of SFA-BTM NPs at concentrations of 6.25 to 200 μg mL^-1^. The pure water and PBS were used as positive and negative controls, representing 100% and 0% hemolysis rates, respectively.

1. **Supplementary Tables**

**Table S1.** X-Ray Crystallographic Data of DMA-BTM.

| **CCDC** | **2048329** |
| --- | --- |
| Empirical formula | C_28_H_18_NCl_6_ |
| Formula weight | 581.13 |
| Temperature/K | 98.0 |
| Crystal system | orthorhombic |
| Space group | Pna2_1_ |
| a/Å | 31.980(2) |
| b/Å | 8.5361(6) |
| c/Å | 18.8916(12) |
| α/° | 90 |
| β/° | 90 |
| γ/° | 90 |
| Volume/Å^3^ | 5157.1(6) |
| Z | 8 |
| ρ_calc_g/cm^3^ | 1.497 |
| μ/mm^‑1^ | 0.686 |
| F(000) | 2360.0 |
| Crystal size/mm^3^ | 0.05 × 0.03 × 0.02 |
| Radiation | MoKα (λ = 0.71073) |
| 2Θ range for data collection/° | 5.008 to 55.156 |
| Index ranges | -41 ≤ h ≤ 41, -11 ≤ k ≤ 11, -22 ≤ l ≤ 24 |
| Reflections collected | 70362 |
| Independent reflections | 11509 [R_int_ = 0.0313, R_sigma_ = 0.0213] |
| Data/restraints/parameters | 11509/1/635 |
| Goodness-of-fit on F^2^ | 1.097 |
| Final R indexes [I>=2σ (I)] | R_1_ = 0.0392, wR_2_ = 0.0831 |
| Final R indexes [all data] | R_1_ = 0.0436, wR_2_ = 0.0849 |
| Largest diff. peak/hole / e Å^-3^ | 0.25/-0.26 |
| Flack parameter | 0.119(10) |

**Table S2.** X-Ray Crystallographic Data of DPA-BTM.

| **CCDC** | **2009653** |
| --- | --- |
| Empirical formula | C_38_H_22_NCl_6_ |
| Formula weight | 705.26 |
| Temperature/K | 293.0 |
| Crystal system | monoclinic |
| Space group | P2_1_/c |
| a/Å | 16.2912(6) |
| b/Å | 9.8491(4) |
| c/Å | 21.3002(8) |
| α/° | 90 |
| β/° | 109.2020(10) |
| γ/° | 90 |
| Volume/Å^3^ | 3227.6(2) |
| Z | 4 |
| ρ_calc_g/cm^3^ | 1.451 |
| μ/mm^‑1^ | 0.562 |
| F(000) | 1436.0 |
| Crystal size/mm^3^ | 0.05 × 0.03 × 0.02 |
| Radiation | MoKα (λ = 0.71073) |
| 2Θ range for data collection/° | 5.296 to 54.97 |
| Index ranges | -21 ≤ h ≤ 21, -12 ≤ k ≤ 12, -27 ≤ l ≤ 24 |
| Reflections collected | 59630 |
| Independent reflections | 7395 [R_int_ = 0.0427, R_sigma_ = 0.0250] |
| Data/restraints/parameters | 7395/0/406 |
| Goodness-of-fit on F^2^ | 1.102 |
| Final R indexes [I>=2σ (I)] | R_1_ = 0.0514, wR_2_ = 0.1113 |
| Final R indexes [all data] | R_1_ = 0.0681, wR_2_ = 0.1193 |
| Largest diff. peak/hole / e Å^-3^ | 0.71/-0.44 |

**Table S3.** X-Ray Crystallographic Data of SFA-BTM.

| **CCDC** | **2083410** |
| --- | --- |
| Empirical formula | C_38_H_20_NCl_6_ |
| Formula weight | 703.25 |
| Temperature/K | 100.0 |
| Crystal system | triclinic |
| Space group | P-1 |
| a/Å | 8.1791(8) |
| b/Å | 19.826(2) |
| c/Å | 21.356(2) |
| α/° | 106.398(4) |
| β/° | 90.444(3) |
| γ/° | 95.487(4) |
| Volume/Å^3^ | 3304.7(6) |
| Z | 4 |
| ρ_calc_g/cm^3^ | 1.413 |
| μ/mm^‑1^ | 0.549 |
| F(000) | 1428.0 |
| Crystal size/mm^3^ | 0.05 × 0.03 × 0.02 |
| Radiation | MoKα (λ = 0.71073) |
| 2Θ range for data collection/° | 5.034 to 55.012 |
| Index ranges | -10 ≤ h ≤ 10, -25 ≤ k ≤ 25, -27 ≤ l ≤ 27 |
| Reflections collected | 64736 |
| Independent reflections | 14881 [R_int_ = 0.1276, R_sigma_ = 0.1288] |
| Data/restraints/parameters | 14881/0/811 |
| Goodness-of-fit on F^2^ | 1.148 |
| Final R indexes [I>=2σ (I)] | R_1_ = 0.1118, wR_2_ = 0.2216 |
| Final R indexes [all data] | R_1_ = 0.1888, wR_2_ = 0.2517 |
| Largest diff. peak/hole / e Å^-3^ | 0.57/-0.58 |

**Table S4.** Selected torsion angles [^o^] and bond angles [^o^] of DMA-BTM crystal structure.


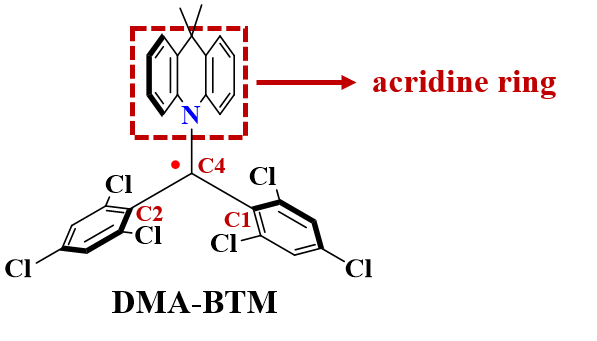


| Bond angles  C1C4N1  C2C4N1  C2C4C1  Torsion angles between C2N1C1 plain and  acridine ring  2,4,6-trichlorophenyl including C1  2,4,6-trichlorophenyl including C2 | Φ(DMA-BTM)  121.0°  116.1°  122.9°  Φ(DMA-BTM)  52.9°  33.3°  51.9° |
| --- | --- |

**Table S5.** Selected torsion angles [^o^] and bond angles [^o^] of DPA-BTM crystal structure.


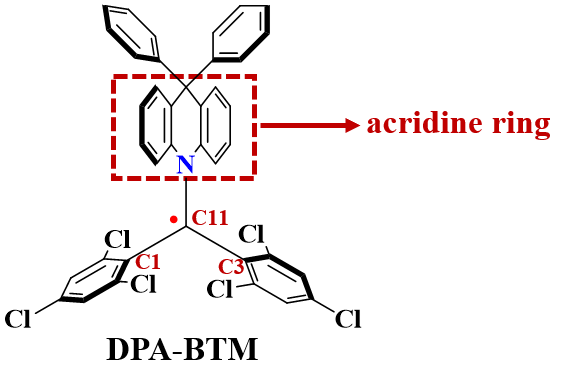


| Bond angles  C1C11C3  N1C11C1  N1C11C3  Torsion angles between C1N1C3 plain and  acridine ring  2,4,6-trichlorophenyl including C1  2,4,6-trichlorophenyl including C3 | Φ(DPA-BTM) |
| --- | --- |
|  | 118.4° |
|  | 118.1° |
|  | 110.2° |
|  | Φ(DPA-BTM) |
|  | 54.3° |
|  | 39.5° |
|  | 50.9° |

**Table S6.** Selected torsion angles [^o^] and bond angles [^o^] of SFA-BTM crystal structure.


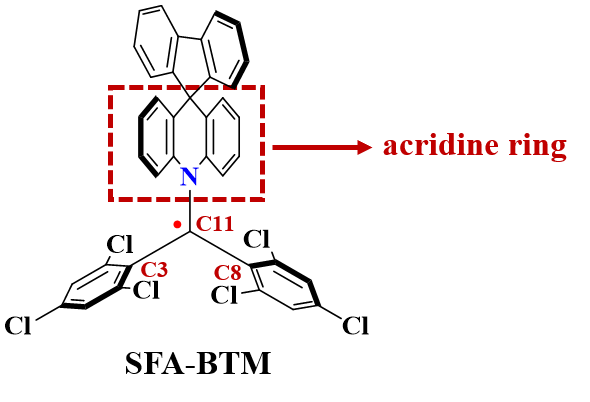


| Bond angles  C8C11C3  C8C11N1  N1C11C3  Torsion angles between C8N1C3 plain and  acridine ring  2,4,6-trichlorophenyl including C8  2,4,6-trichlorophenyl including C3 | Φ(SFA-BTM)  120.3°  120.0°  119.6°  Φ(SFA-BTM)  57.3°  43.2°  51.9° |
| --- | --- |

**Table S7.** The absorption wavelength (λ_EXP_, experimental wavelength, λ_B3LYP_, calculated wavelength), excitation energy of the first excited state (D_1_) and the energy gap (E_gap_) of SOMO and SUMO calculated at B3LYP/6-31+G(d,p) in the cyclohexane phase. MO transition: molecule orbitals transition.

| **Molecules** | **λ_EXP_**  **(nm)** | **λ_B3LYP_**  **(nm)** | **MO transition** | **D_1_ state energy**  **(eV)** | **E_gap_**  **(eV)** |
| --- | --- | --- | --- | --- | --- |
| **DMA-BTM** | 710 | 758 | 147 β → 148 β | 1.636 | 1.87 |
| **DPA-BTM** | 683 | 705 | 179 β → 180 β | 1.759 | 1.94 |
| **SFA-BTM** | 721 | 755 | 178 β → 179 β | 1.642 | 1.89 |

**Table S8.** Excited states of DMA-BTM calculated by TD-DFT method.

Excited State 1: 2.037-A 1.6359 eV 757.89 nm f=0.0897 <S**2>=0.787

147B ->148B 0.99033

This state for optimization and/or second-order correction.

Total Energy, E(TD-HF/TD-DFT) = -3893.70844450

Copying the excited state density for this state as the 1-particle RhoCI density.

Excited State 2: 2.198-A 2.5772 eV 481.09 nm f=0.0251 <S**2>=0.958

147A ->149A -0.20077

148A ->149A 0.81521

141B ->148B 0.25189

144B ->148B -0.22346

145B ->148B -0.28788

146B ->148B -0.14970

147B ->149B 0.12166

Excited State 3: 2.072-A 2.8185 eV 439.89 nm f=0.0224 <S**2>=0.824

148A ->149A 0.21874

148A ->152A 0.11135

145B ->148B 0.10451

146B ->148B 0.93682

Excited State 4: 2.108-A 2.8768 eV 430.98 nm f=0.0089 <S**2>=0.861

147A ->150A -0.13313

148A ->149A -0.18454

148A ->150A 0.81761

143B ->148B -0.32935

145B ->148B -0.32838

146B ->148B 0.12487

Excited State 5: 2.160-A 2.9138 eV 425.51 nm f=0.0614 <S**2>=0.916

148A ->149A 0.35005

148A ->150A 0.37340

144B ->148B 0.40693

145B ->148B 0.68479

146B ->148B -0.17041

Excited State 6: 2.174-A 3.0052 eV 412.56 nm f=0.0020 <S**2>=0.932

148A ->151A -0.45297

148A ->154A -0.14569

148A ->155A -0.13150

142B ->148B 0.26360

144B ->148B 0.65087

145B ->148B -0.44210

Excited State 7: 2.118-A 3.0345 eV 408.59 nm f=0.0037 <S**2>=0.871

147A ->151A -0.12872

148A ->150A -0.10017

148A ->151A 0.68765

142B ->148B -0.41865

143B ->148B 0.13520

144B ->148B 0.41045

145B ->148B -0.29078

Excited State 8: 2.129-A 3.1443 eV 394.32 nm f=0.0185 <S**2>=0.883

148A ->150A 0.36604

140B ->148B -0.15905

141B ->148B -0.12810

143B ->148B 0.86674

Excited State 9: 2.347-A 3.1594 eV 392.43 nm f=0.0498 <S**2>=1.127

141A ->149A -0.12761

147A ->149A -0.12460

147A ->152A -0.10576

148A ->149A -0.22056

148A ->151A 0.10829

148A ->152A 0.28995

148A ->156A -0.16842

140B ->148B 0.41766

141B ->148B 0.53022

141B ->149B 0.10748

142B ->148B 0.28503

143B ->148B 0.26135

145B ->148B 0.11374

146B ->148B -0.13301

Excited State 10: 2.387-A 3.2033 eV 387.05 nm f=0.0398 <S**2>=1.175

147A ->149A -0.13293

148A ->149A -0.17861

148A ->151A -0.26840

148A ->154A -0.16515

148A ->155A -0.16928

140B ->148B -0.29563

141B ->148B 0.52820

142B ->148B -0.50300

147B ->154B 0.11786

Excited State 11: 2.306-A 3.2386 eV 382.83 nm f=0.0115 <S**2>=1.080

148A ->151A 0.43253

148A ->152A -0.23405

148A ->156A 0.12847

140B ->148B -0.39532

141B ->148B 0.18878

142B ->148B 0.60522

Excited State 12: 2.825-A 3.4022 eV 364.43 nm f=0.0361 <S**2>=1.745

146A ->157A 0.12273

147A ->149A -0.17030

147A ->154A 0.12995

147A ->155A 0.10677

148A ->152A -0.18661

148A ->153A -0.13545

148A ->154A 0.44403

148A ->155A 0.35563

141B ->148B 0.27437

144B ->148B 0.38677

146B ->156B -0.11163

147B ->149B 0.20207

147B ->150B 0.17962

147B ->154B -0.27035

147B ->155B -0.16570

Excited State 13: 2.955-A 3.4804 eV 356.24 nm f=0.0213 <S**2>=1.933

143A ->151A 0.10922

147A ->149A -0.28286

148A ->152A 0.51617

148A ->154A -0.10607

140B ->148B -0.11233

141B ->148B -0.32399

143B ->151B -0.10045

147B ->149B 0.57082

147B ->152B -0.12966

Excited State 14: 2.816-A 3.5318 eV 351.05 nm f=0.0214 <S**2>=1.733

147A ->149A 0.20817

148A ->152A 0.59169

148A ->155A 0.21646

148A ->156A 0.11589

140B ->148B -0.26822

141B ->148B 0.24047

147B ->149B -0.44964

147B ->152B -0.12722

147B ->155B -0.11747

Excited State 15: 2.125-A 3.6613 eV 338.63 nm f=0.0064 <S**2>=0.878

147A ->149A -0.10632

147A ->153A -0.12332

148A ->153A 0.95262

148A ->154A 0.10808

148A ->155A 0.11268

Excited State 16: 2.853-A 3.6714 eV 337.70 nm f=0.0127 <S**2>=1.785

144A ->154A 0.12936

144A ->155A 0.14004

145A ->157A 0.16626

146A ->152A -0.14832

146A ->156A -0.11388

148A ->154A -0.43285

148A ->155A 0.50195

148A ->156A 0.31906

148A ->162A -0.12877

144B ->154B -0.15960

144B ->155B -0.12782

145B ->156B -0.15935

146B ->152B 0.15037

146B ->155B 0.11723

147B ->151B -0.10249

147B ->157B 0.17198

Excited State 17: 2.942-A 3.7379 eV 331.69 nm f=0.0019 <S**2>=1.914

142A ->151A -0.14219

143A ->150A -0.15381

144A ->154A 0.10614

144A ->155A 0.11518

145A ->157A 0.16692

146A ->152A -0.12152

146A ->156A -0.10641

147A ->150A -0.10505

148A ->152A 0.31021

148A ->154A 0.27539

148A ->155A -0.33539

148A ->156A 0.21515

148A ->162A -0.16358

140B ->148B 0.10374

142B ->151B 0.13539

143B ->150B 0.14600

144B ->154B -0.12639

145B ->156B -0.16637

146B ->152B 0.14406

147B ->150B 0.35147

147B ->152B 0.12084

Excited State 18: 2.976-A 3.7588 eV 329.85 nm f=0.0157 <S**2>=1.964

141A ->149A 0.11498

142A ->151A -0.26875

143A ->150A -0.29680

147A ->149A -0.10421

148A ->155A 0.19481

140B ->148B 0.48127

141B ->149B -0.13565

142B ->151B 0.24289

143B ->150B 0.27299

147B ->149B -0.17097

147B ->150B -0.43186

147B ->152B -0.12445

Excited State 19: 2.518-A 3.7646 eV 329.35 nm f=0.1258 <S**2>=1.335

142A ->150A -0.24526

143A ->150A -0.10411

143A ->151A -0.19952

147A ->149A 0.54188

140B ->148B 0.11494

141B ->148B 0.22190

142B ->150B 0.20071

143B ->151B 0.17371

147B ->149B 0.59148

Excited State 20: 2.952-A 3.7726 eV 328.65 nm f=0.0125 <S**2>=1.929

142A ->151A -0.10720

143A ->150A -0.11335

147A ->150A -0.17191

148A ->154A -0.38431

148A ->155A 0.19585

148A ->156A -0.16238

140B ->148B 0.17036

142B ->151B 0.10375

143B ->150B 0.10946

147B ->150B 0.71654

**Table S9.** Excited states of DPA-BTM calculated by TD-DFT method.

Excited State 1: 2.041-A 1.7585 eV 705.07 nm f=0.0913 <S**2>=0.792

179B ->180B 0.98898

This state for optimization and/or second-order correction.

Total Energy, E(TD-HF/TD-DFT) = -4277.18723314

Copying the excited state density for this state as the 1-particle RhoCI density.

Excited State 2: 2.211-A 2.6076 eV 475.46 nm f=0.0294 <S**2>=0.972

179A ->181A 0.18997

180A ->181A 0.82450

169B ->180B 0.20906

171B ->180B 0.12459

173B ->180B 0.21422

175B ->180B -0.23648

178B ->180B 0.15451

179B ->181B -0.10670

Excited State 3: 2.053-A 2.7821 eV 445.65 nm f=0.0074 <S**2>=0.804

180A ->181A -0.16946

171B ->180B 0.12852

172B ->180B 0.10187

178B ->180B 0.95639

Excited State 4: 2.112-A 2.8900 eV 429.01 nm f=0.0178 <S**2>=0.865

179A ->182A 0.11302

180A ->181A -0.26691

180A ->182A 0.53857

180A ->183A 0.12234

170B ->180B 0.23337

172B ->180B 0.16434

173B ->180B 0.19048

175B ->180B -0.50263

177B ->180B 0.42708

Excited State 5: 2.164-A 2.9170 eV 425.04 nm f=0.0169 <S**2>=0.921

180A ->181A 0.10383

180A ->182A 0.69214

180A ->184A 0.10051

168B ->180B 0.13389

170B ->180B 0.16379

172B ->180B 0.16538

175B ->180B 0.48455

176B ->180B 0.11430

177B ->180B -0.30319

Excited State 6: 2.121-A 2.9609 eV 418.74 nm f=0.0169 <S**2>=0.875

180A ->181A 0.19851

180A ->183A -0.17354

172B ->180B 0.16578

173B ->180B -0.23097

174B ->180B 0.28117

175B ->180B 0.32865

176B ->180B 0.22916

177B ->180B 0.74306

Excited State 7: 2.137-A 3.0617 eV 404.95 nm f=0.0104 <S**2>=0.892

179A ->183A 0.11499

180A ->181A 0.16245

180A ->183A 0.57443

169B ->180B -0.11698

170B ->180B 0.40003

171B ->180B 0.15640

172B ->180B -0.41192

173B ->180B -0.13878

174B ->180B 0.41727

Excited State 8: 2.271-A 3.1388 eV 395.00 nm f=0.0146 <S**2>=1.039

180A ->182A -0.22198

180A ->183A 0.54685

180A ->185A 0.17858

180A ->188A 0.12062

180A ->189A -0.12077

168B ->180B 0.40921

171B ->180B -0.14288

172B ->180B 0.22728

174B ->180B -0.30198

175B ->180B 0.20168

176B ->180B -0.11272

177B ->180B 0.23371

Excited State 9: 2.265-A 3.1665 eV 391.55 nm f=0.0966 <S**2>=1.032

179A ->181A 0.12500

180A ->181A -0.28759

180A ->182A -0.13947

180A ->184A 0.18391

168B ->180B 0.18977

169B ->180B 0.50183

171B ->180B 0.33983

173B ->180B 0.27362

174B ->180B 0.24232

176B ->180B 0.41390

178B ->180B -0.10050

Excited State 10: 2.146-A 3.1819 eV 389.66 nm f=0.0158 <S**2>=0.901

180A ->182A -0.29330

180A ->185A -0.10776

168B ->180B -0.23615

170B ->180B 0.50776

171B ->180B -0.16402

172B ->180B 0.54066

173B ->180B 0.19066

176B ->180B 0.33661

177B ->180B -0.18293

Excited State 11: 2.135-A 3.2103 eV 386.21 nm f=0.0094 <S**2>=0.889

180A ->182A 0.16280

180A ->183A 0.22865

180A ->184A -0.10318

169B ->180B -0.20068

170B ->180B -0.38591

171B ->180B -0.12672

172B ->180B -0.14472

174B ->180B -0.25176

175B ->180B -0.10992

176B ->180B 0.74467

Excited State 12: 2.342-A 3.2361 eV 383.12 nm f=0.0193 <S**2>=1.121

180A ->181A 0.10811

180A ->183A -0.32169

180A ->185A 0.18191

180A ->186A 0.14131

180A ->189A -0.11066

168B ->180B 0.41854

169B ->180B -0.20399

170B ->180B 0.18171

171B ->180B -0.22598

173B ->180B -0.19680

174B ->180B 0.31705

175B ->180B -0.36893

176B ->180B 0.16928

177B ->180B -0.14678

Excited State 13: 2.082-A 3.2790 eV 378.12 nm f=0.0033 <S**2>=0.834

180A ->183A 0.34826

170B ->180B -0.48191

171B ->180B -0.12100

172B ->180B 0.49478

173B ->180B -0.21041

174B ->180B 0.45506

175B ->180B -0.14374

176B ->180B -0.13647

177B ->180B -0.22609

Excited State 14: 2.695-A 3.4237 eV 362.13 nm f=0.0241 <S**2>=1.565

179A ->188A 0.10843

180A ->184A 0.45054

180A ->188A -0.34029

180A ->190A 0.13075

169B ->180B -0.15513

173B ->180B -0.26530

174B ->180B -0.34379

175B ->180B -0.32111

179B ->182B 0.12281

179B ->184B -0.17881

179B ->188B -0.15985

Excited State 15: 2.315-A 3.4984 eV 354.40 nm f=0.0062 <S**2>=1.090

179A ->181A -0.14202

180A ->184A -0.30785

169B ->180B -0.19036

171B ->180B 0.76700

172B ->180B 0.25506

173B ->180B -0.12900

174B ->180B -0.14624

178B ->180B -0.13432

179B ->181B 0.19965

Excited State 16: 2.625-A 3.5111 eV 353.12 nm f=0.0039 <S**2>=1.473

172A ->183A -0.10491

179A ->181A -0.25163

180A ->185A -0.24649

180A ->188A -0.16988

169B ->180B -0.15952

170B ->180B -0.16654

171B ->180B -0.16999

172B ->183B -0.10013

173B ->180B 0.66000

174B ->180B 0.12720

179B ->181B 0.34547

Excited State 17: 2.381-A 3.5183 eV 352.40 nm f=0.0166 <S**2>=1.168

180A ->184A -0.34916

180A ->185A -0.43822

169B ->180B 0.52816

171B ->180B -0.22223

172B ->180B -0.12636

173B ->180B -0.31950

174B ->180B -0.21407

178B ->180B 0.11026

179B ->181B 0.18678

179B ->185B -0.12746

Excited State 18: 2.911-A 3.5916 eV 345.21 nm f=0.0125 <S**2>=1.868

170A ->182A 0.12063

172A ->183A -0.12033

179A ->181A -0.29896

180A ->181A 0.11626

180A ->184A 0.18781

180A ->185A 0.42217

180A ->186A -0.13958

180A ->188A 0.10510

168B ->180B -0.23439

169B ->180B 0.39186

170B ->182B -0.12470

172B ->183B -0.11323

179B ->181B 0.47261

179B ->185B 0.10984

Excited State 19: 2.886-A 3.6090 eV 343.54 nm f=0.0434 <S**2>=1.832

175A ->184A -0.12092

177A ->184A -0.11370

178A ->188A -0.12262

179A ->197A -0.11378

180A ->184A -0.13812

180A ->186A 0.63729

180A ->187A -0.17268

180A ->188A -0.10114

180A ->191A 0.14453

180A ->197A 0.18172

168B ->180B -0.11550

175B ->184B 0.10159

177B ->185B 0.12296

178B ->188B 0.11340

179B ->186B 0.10176

179B ->191B 0.12564

179B ->200B -0.11450

Excited State 20: 3.450-A 3.6675 eV 338.06 nm f=0.0016 <S**2>=2.726

171A ->188A -0.12204

171A ->190A -0.13310

173A ->184A -0.11125

173A ->193A -0.11688

176A ->188A -0.11507

176A ->190A -0.14307

178A ->184A 0.17871

178A ->185A -0.21278

178A ->188A 0.20948

178A ->189A 0.10594

178A ->190A 0.25533

178A ->192A -0.10381

171B ->188B 0.11973

171B ->189B -0.14209

173B ->184B -0.15226

173B ->192B -0.11324

173B ->193B 0.11110

176B ->189B -0.13097

178B ->184B 0.24833

178B ->185B 0.15404

178B ->188B -0.18866

178B ->189B 0.25230

178B ->192B 0.10426

**Table S10.** Excited states of SFA-BTM calculated by TD-DFT method.

Excited State 1: 2.037-A 1.6415 eV 755.31 nm f=0.0987 <S**2>=0.788

178B ->179B 0.99047

This state for optimization and/or second-order correction.

Total Energy, E(TD-HF/TD-DFT) = -4276.00621264

Copying the excited state density for this state as the 1-particle RhoCI density.

Excited State 2: 2.045-A 2.2872 eV 542.07 nm f=0.0001 <S**2>=0.795

176B ->179B -0.15074

177B ->179B 0.98445

Excited State 3: 2.211-A 2.6021 eV 476.49 nm f=0.0187 <S**2>=0.972

177A ->180A 0.20676

179A ->180A 0.78474

170B ->179B 0.25763

173B ->179B -0.38262

176B ->179B -0.18542

178B ->180B 0.11995

Excited State 4: 2.067-A 2.7968 eV 443.31 nm f=0.0124 <S**2>=0.819

179A ->184A 0.11081

175B ->179B 0.97463

Excited State 5: 2.052-A 2.8316 eV 437.85 nm f=0.0237 <S**2>=0.802

179A ->180A 0.29558

179A ->181A -0.10175

169B ->179B -0.10361

173B ->179B 0.13152

176B ->179B 0.91398

177B ->179B 0.14129

Excited State 6: 2.126-A 2.9190 eV 424.75 nm f=0.0010 <S**2>=0.880

177A ->181A 0.14210

179A ->181A 0.85686

172B ->179B -0.42305

176B ->179B 0.16048

Excited State 7: 2.215-A 2.9373 eV 422.11 nm f=0.0535 <S**2>=0.977

179A ->180A 0.38237

179A ->181A 0.11537

179A ->186A 0.18454

173B ->179B 0.82437

176B ->179B -0.22572

178B ->186B -0.11342

Excited State 8: 3.473-A 3.0220 eV 410.27 nm f=0.0000 <S**2>=2.766

168A ->204A -0.12217

174A ->192A 0.13814

176A ->183A -0.21202

176A ->185A -0.10923

178A ->182A -0.10859

178A ->183A -0.57884

178A ->185A 0.17835

167B ->204B 0.12280

174B ->192B 0.13660

176B ->182B 0.21770

177B ->182B 0.58130

177B ->184B -0.12450

177B ->185B -0.14866

Excited State 9: 2.122-A 3.0389 eV 407.98 nm f=0.0005 <S**2>=0.876

177A ->182A 0.14615

179A ->182A 0.73627

179A ->183A -0.11664

171B ->179B -0.61642

Excited State 10: 2.100-A 3.1417 eV 394.64 nm f=0.0198 <S**2>=0.852

179A ->181A 0.44780

172B ->179B 0.87283

Excited State 11: 2.397-A 3.1665 eV 391.55 nm f=0.0028 <S**2>=1.187

170A ->180A 0.16095

171A ->182A -0.11589

172A ->181A 0.11977

173A ->180A -0.13951

177A ->184A 0.12706

177A ->189A -0.14087

179A ->182A -0.21426

179A ->184A 0.29959

179A ->189A -0.24026

168B ->179B 0.62263

170B ->180B -0.13434

171B ->179B -0.37300

171B ->183B -0.12128

172B ->181B 0.13187

173B ->180B 0.11780

174B ->179B -0.10210

175B ->179B -0.16699

Excited State 12: 2.444-A 3.1684 eV 391.31 nm f=0.1017 <S**2>=1.244

177A ->180A 0.15666

177A ->186A -0.11920

179A ->180A -0.31367

179A ->186A 0.30178

169B ->179B -0.20817

170B ->179B 0.74869

178B ->186B -0.16709

Excited State 13: 2.143-A 3.2319 eV 383.62 nm f=0.0077 <S**2>=0.898

179A ->182A 0.53297

179A ->184A 0.12803

168B ->179B 0.23084

171B ->179B 0.59358

174B ->179B -0.48053

Excited State 14: 2.092-A 3.2468 eV 381.87 nm f=0.0000 <S**2>=0.844

179A ->182A 0.24796

179A ->184A 0.13256

168B ->179B 0.18965

171B ->179B 0.30256

174B ->179B 0.86828

Excited State 15: 2.802-A 3.3838 eV 366.41 nm f=0.0362 <S**2>=1.713

175A ->190A 0.12851

177A ->180A -0.14980

177A ->186A -0.15545

179A ->186A 0.59855

179A ->192A -0.10139

169B ->179B 0.15428

170B ->179B -0.33707

173B ->179B -0.34615

175B ->189B -0.11957

178B ->180B -0.14530

178B ->181B -0.16163

178B ->186B -0.30451

Excited State 16: 2.235-A 3.4343 eV 361.01 nm f=0.0000 <S**2>=0.999

179A ->182A 0.14877

179A ->183A 0.97951

Excited State 17: 2.597-A 3.4777 eV 356.51 nm f=0.0158 <S**2>=1.436

177A ->184A -0.14471

179A ->184A 0.80560

179A ->185A -0.21409

179A ->188A 0.17186

179A ->189A 0.12623

168B ->179B -0.24557

178B ->184B -0.18625

178B ->185B 0.14375

Excited State 18: 3.227-A 3.5137 eV 352.86 nm f=0.0097 <S**2>=2.354

171A ->181A 0.15521

172A ->182A -0.14494

177A ->180A 0.39709

179A ->180A -0.15572

170B ->179B -0.36736

171B ->181B -0.14790

172B ->183B 0.13931

178B ->180B 0.70339

Excited State 19: 3.090-A 3.6305 eV 341.51 nm f=0.0196 <S**2>=2.138

169A ->190A 0.13063

170A ->186A 0.11624

173A ->186A 0.16518

175A ->184A 0.21497

175A ->185A -0.11815

175A ->188A 0.14087

175A ->189A 0.11472

176A ->190A -0.16454

177A ->198A 0.12533

178A ->190A 0.13530

179A ->185A -0.28811

179A ->188A 0.45670

179A ->189A 0.25358

179A ->198A -0.18378

169B ->189B -0.12572

170B ->186B -0.11691

173B ->186B -0.16428

175B ->184B -0.18955

175B ->185B 0.18716

175B ->188B 0.11755

176B ->186B 0.10314

176B ->189B 0.16047

177B ->186B -0.10373

177B ->189B -0.13296

178B ->190B -0.15216

178B ->200B 0.14163

Excited State 20: 2.142-A 3.6784 eV 337.06 nm f=0.0027 <S**2>=0.897

179A ->187A 0.18247

179A ->190A 0.10380

169B ->179B 0.86733

170B ->179B 0.28553

176B ->179B 0.13481

178B ->180B 0.20828

**Table S11.** The oxidation and reduction potentials of DMA-BTM and SFA-BTM and their corresponding energy levels.

|  | **E_Ox._/ V** | **E _SOMO_/ eV** | **E_Red._/ V** | **E _SUMO_/ eV** |
| --- | --- | --- | --- | --- |
| DMA-BTM | -0.20 | -4.60 | -1.11 | -3.69 |
| SFA-BTM | -0.02 | -4.78 | -1.01 | -3.79 |

**Table S12.** The oxidation and reduction potentials of DPA-BTM and its corresponding energy levels.

|  | **E_Ox1._/ V** | **E_SOMO_/ eV** | **E_Ox2._/ V** | **E_178β_/ eV** | **E_Red._/ V** | **E_SUMO_/ eV** |
| --- | --- | --- | --- | --- | --- | --- |
| DPA-BTM | -0.13 | -4.67 | 0.73 | -5.53 | -1.05 | -3.75 |

**Table S13.** The internal conversion (IC) coefficient calculated by the Molecular Materials Property Prediction (MOMAP) program.

|  | **DMA-BTM** | **DPA-BTM** | **SFA-BTM** |
| --- | --- | --- | --- |
| E(D_0_) | -3893.72925592 | -4277.19631177 | -4276.01414725 |
| E(D_1_) | -3893.68587439 | -4277.15178415 | -4275.97025136 |
| E(ad) | 0.04338 | 0.04453 | 0.04390 |
| K_IC_(S^-1^) | 6.0098×10^11^ | 6.78×10^11^ | 1.4438×10^12^ |

**Table S14.** Summary of recently reported organic radicals as photothermal agents.

| **Agents** | **Types** | **Stability** | **IC coefficient (S^-1^)** | **NIR-II FL** | **PCE (%)** | **Ref.** |
| --- | --- | --- | --- | --- | --- | --- |
| SFA-BTM NPs | neutral π-radical | good | 1.4438×10^12^ | yes | 49.0 | This work |
| TTF-(TTF^+•^)_2_-RC | radical cation | poor | - | - | 62.9 | *Adv. Sci.* **2023***, 10,* 2300980. |
| (NDI-2CB[7])^•−^ | radical anion | poor | - | - | 66.9 | *Angew. Chem. Int. Ed.* **2023***, 62,* e202308513. |
| PDI-2CB[7] | radical anion | poor | - | - | 35.7 | *J. Am. Chem. Soc.* **2022***, 144,* 2360. |
| CR-NPs | neutral radical | good | - | - | 78.9 | *J. Am. Chem. Soc.* **2022***, 144,* 3458. |
| 2MPT^•+^-CB[8] | radical cation | poor | - | - | 54.6 | *Angew. Chem. Int. Ed.* ***2019****, 58,* 15526. |
| Py-BPy^+•^-COF/PEG | radical cation | poor | - | - | 63.8/55.2 | *J. Am. Chem. Soc.***2019***, 141,* 14433. |
| Zr-PDI^•−^ | radical anion | poor | - | - | 52.3 | *Nat. Commun.* ***2019****, 10,* 767. |
| CPPDI | radical anion | poor | - | - | - | *Angew. Chem. Int. Ed.****2017****, 56,* 16239*.* |

**4. References**

[1] S. N. Bagriantsev, K.-H. Ang, A. Gallardo-Godoy, K. A. Clark, M. R. Arkin, A. R. Renslo, D. L. Minor, Jr., *ACS Chem. Biol.* **2013**, *8*, 1841.

[2] X. Ai, Y. Chen, Y. Feng, F. Li, *Angew. Chem. Int. Ed.* **2018**, *57*, 2869.

[3] X. Liu, F. Liang, Y. Yuan, L. Cui, Z. Jiang, L. Liao, *Chem. Commun.* **2016**, *52*, 8149.

[4] Y. Niu, W. Li, Q. Peng, H. Geng, Y. Yi, L. Wang, G. Nan, D. Wang, Z. Shuai, *Mol. Phys.* **2018**, *116*, 1078.

[5] B. Tang, W. Li, Y. Chang, B. Yuan, Y. Wu, M. Zhang, J. Xu, J. Li, X. Zhang, *Angew. Chem. Int. Ed.* **2019**, *58*, 15526.

[6] W. N. Arifin, W. M. Zahiruddin, *Malays. J. Med. Sci.* **2017**, *24*, 101.

[7] H. H. Luu, Q. Kang, J. K. Park, W. Si, Q. Luo, W. Jiang, H. Yin, A. G. Montag, M. A. Simon, T. D. Peabody, R. C. Haydon, C. W. Rinker-Schaeffer, T. He, *Clin. Exp. Metastasis* **2005**, *22*, 319.

[8] Q. Wu, D. Deng, J. Zhang, W. Zou, Y. Yang, Z. Wang, H. Li, R. Zhou, K. Lu, Z. Wei, *Sci. China Chem.* **2019**, *62*, 837.
